# Supplementary material for: WIPI3 and WIPI4 β-propellers are scaffolds for LKB1-AMPK-TSC signalling circuits in the control of autophagy
Source: Nat Commun. 2017 May 31;8:15637. doi: 10.1038/ncomms15637 (PMC5460038; doi:10.1038/ncomms15637)
Supplement: Supplementary Information — Supplementary Figures, Supplementary Note, and Supplementary References [file ncomms15637-s1.pdf]

Supplementary Figure 1

a

```
CLUSTAL multiple sequence alignment by MUSCLE (3.8)


WIPI1           MEAEAA---DAPPGGVESALSCFSFNQDCTSLATGTKAGYKLFSLSSVEQLDQVHGSNEI
WIPI2B          MNLAS----QSGEAGAGQLLFA-NFNQDNTSLAVGSKSGYKFFSLSSVDKLEQIYECTDT
WIPI3           MDSGAMNLLPCNPHGNGLLYA--GFNQDHGCFACGMENGFRVYNTDPLKEKEKQ-EFLE-
WIPI4           MT-------QQPLRGVTSL----RFNQDQSCFCCAMETGVRIYNVEPLMEKGHL-DHEQV
                *             *         ****  .:. . : * ..:. ..: :  :     :

WIPI1           PDVYIVERLFSSSLVVVV-SHTKPR--QMNVYHFK--------KGTEICNYSYSSNILSI
WIPI2B          EDVCIVERLFSSSLVAIV-SLKAPR--KLKVCHFK--------KGTEICNYSYSNTILAV
WIPI3           GGVGHVEMLFRCNYLALVGGGKKPKYPPNKVMIWDDL------KKKTVIEIEFSTEVKAV
WIPI4           GSMGLVEMLHRSNLLALVGGGSSPKFSEISVLIWDDAREGKDSKEKLVLEFTFTKPVLSV
                 .:  ** *. .. :.:* . . *.    .*  :.         * . : :  ::. : ::

WIPI1           RLNRQRLLVCLEESIYIHNIKDMKLLKTLLDIPANPTGLCALSINHSNSYLAYPGSLTSG
WIPI2B          KLNRQRLIVCLEESLYIHNIRDMKVLHTIRETPPNPAGLCALSINNDNCYLAYPGSATIG
WIPI3           KLRRDRIVVVLDSMIKVFTFTHNPHQLHVFETCYNPKGLCVLCPNSNNSLLAFPGTHT-G
WIPI4           RMRHDKIVIVLKNRIYVYSFPDNPRKLFEFDTRDNPKGLCDLCPSLEKQLLVFPGHKC-G
                .:..:.::: *.. : :..:           :   ** *** *. . .:  *.:**    *

WIPI1           EIVLYDGNSLK-----TVCTIAAHEGTLAAITFNASGSKLASASEKGTVIRVFSVPDGQK
WIPI2B          EVQVFDTINLR-----AANMIPAHDSPLAALAFDASGTKLATASEKGTVIRVFSIPEGQK
WIPI3           HVQLVDLASTE----KPPVDIPAHEGVLSCIALNLQGTRIATASEKGTLIRIFDTSSGHL
WIPI4           SLQLVDLASTKPGTSSAPFTINAHQSDIACVSLNQPGTVVASASQKGTLIRLFDTQSKEK
                 : : *   .       .   * **:. ::.::::  *: :*:**:***:**:*.  .

WIPI1           LYEFRRGMKRYVTISSLVFSMDSQFLCASSNTETVHIFKLEQVTNSRPEEPSTWSGYMGK
WIPI2B          LFEFRRGVKRCVSICSLAFSMDGMFLSASSNTETVHIFKLETVKEKPPEEPTTWTGYFGK
WIPI3           IQELRRGSQA-ANIYCINFNQDASLICVSSDHGTAHIFAAEDPKRNKQSS----------
WIPI4           LVELRRGTDP-ATLYCINFSHDSSFLCASSDKGTVHIFALKDTRLNRRSA----------
                : *:*** .   ..: .: *. *. ::..**:  *.***  :     .  .

WIPI1           MFMAATNYLPTQVSDMMHQDRAFATARLNFSGQRNICTLS--TIQKLPRLLVASSSGHLY
WIPI2B          VLMASTSYLPSQVTEMFNQGRAFATVRLPFCGHKNICSLA--TIQKIPRLLVGAADGYLY
WIPI3           --LASASFLP----KYFSSKWSFSKFQVP-SGSPCICAFG----TEPNAVIAICADGSYY
WIPI4           --LARVGKVGPMIGQYVDSQWSLASYTAP-AESACICAFGRNTSKNVNSVIAICVDGTFH
                  :* .. :      . . . .:::.      .     **::.      :    ::. . .*  :

WIPI1           MYNLDPQDGGECVLIKTHSLLGS-GTTEE--NKENDLRPSLPQSYAATVARPS-ASSAST
WIPI2B          MYNLDPQEGGECALMKQHRLDGSLETTNEILDSASHDCPLVTQTYGAAAGKGTYVPSSPT
WIPI3           KFLFNPK-----------------------------------------------------
WIPI4           KYVFTPD-----------------------------------------------------
                 : : *.

WIPI1           VPGYSEDGGALRGEVIPEHEFATGPVCLDDENEFPPIILCRGNQKGKTKQS
WIPI2B          RLAYTDDLGAVGGACLEDEASA---LRLDEDSEHPPMILRTD---------
WIPI3           ------------GECIRDVYAQFLELTDDKL--------------------
WIPI4           ------------GNCNREAFDVYLDICDDDDF-------------------
                            *     :        :  *.
```

```
WIPI3 WT            226-IQELRRGSQA-235
WIPI3 R230A/R231A  226-IQELAAGSQA-235
```

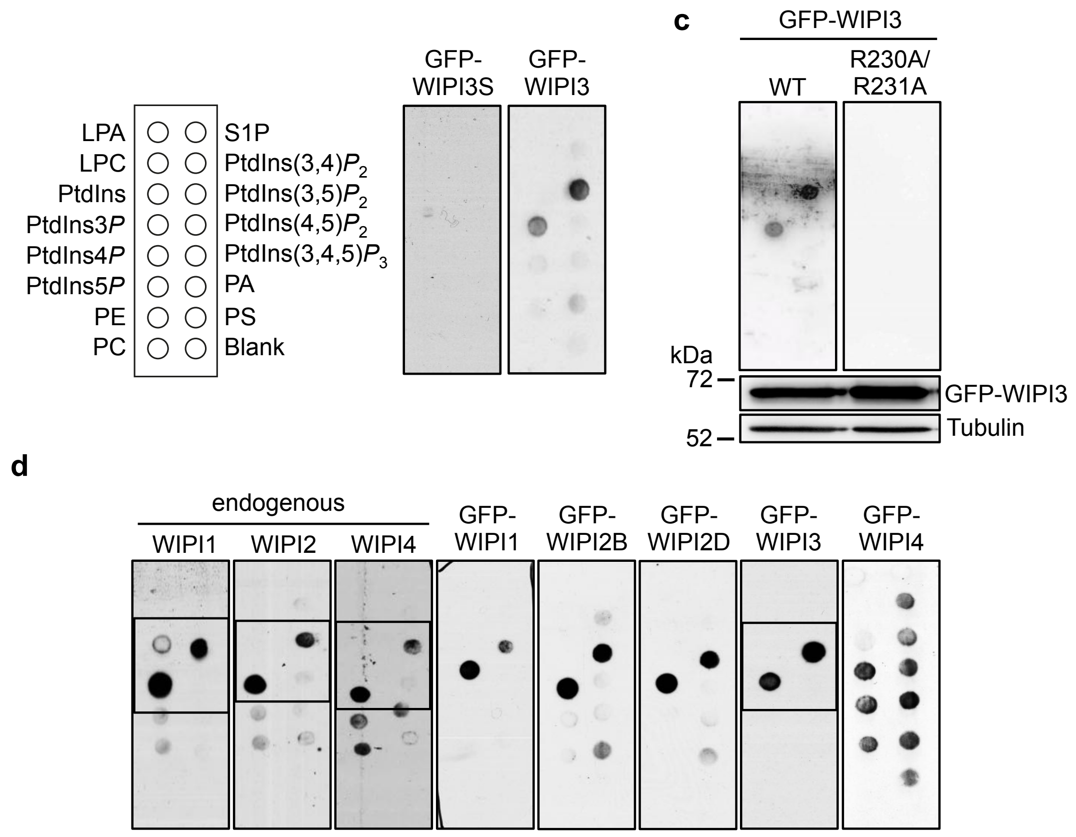

Supplementary Figure 1

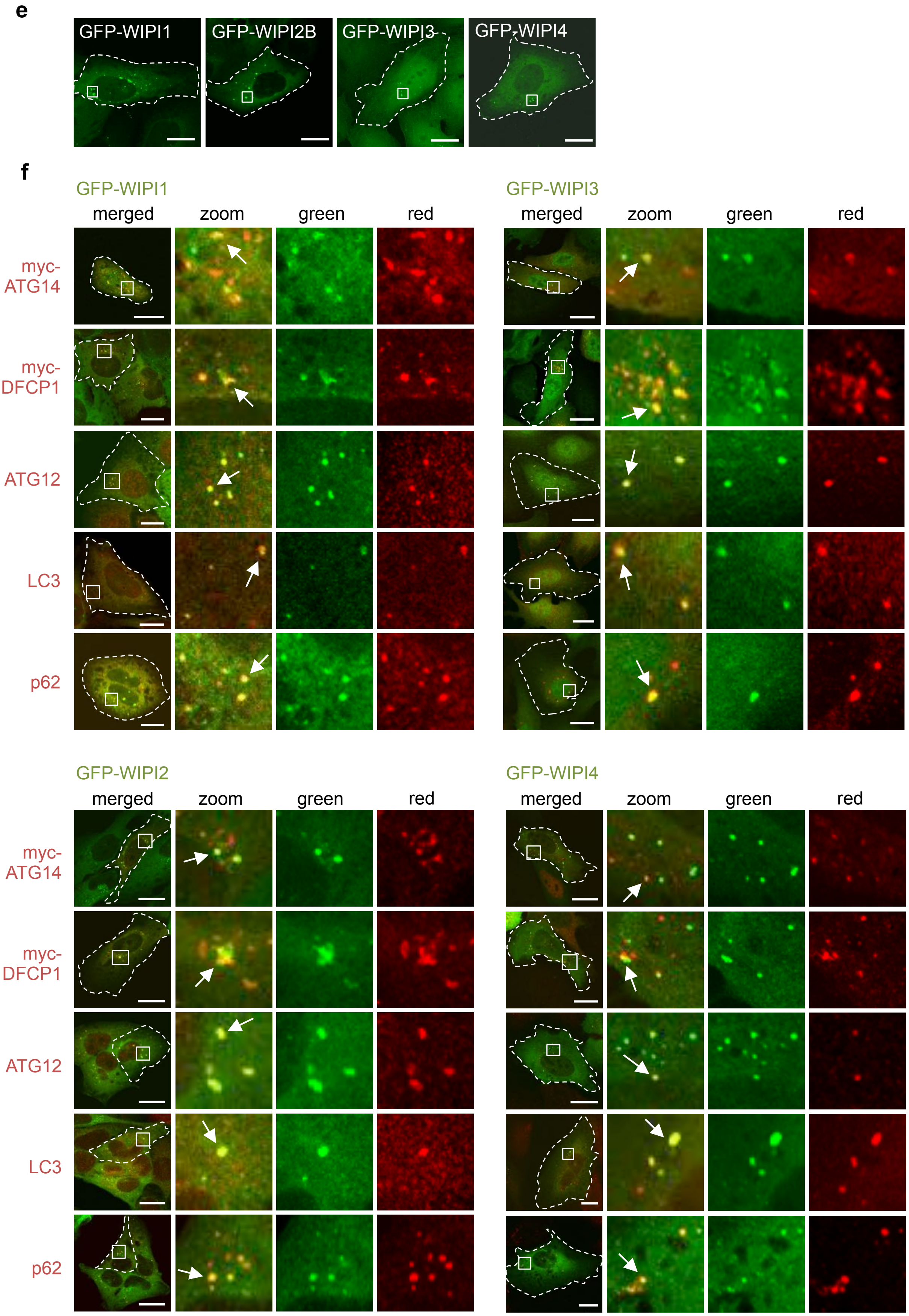

Supplementary Figure 1

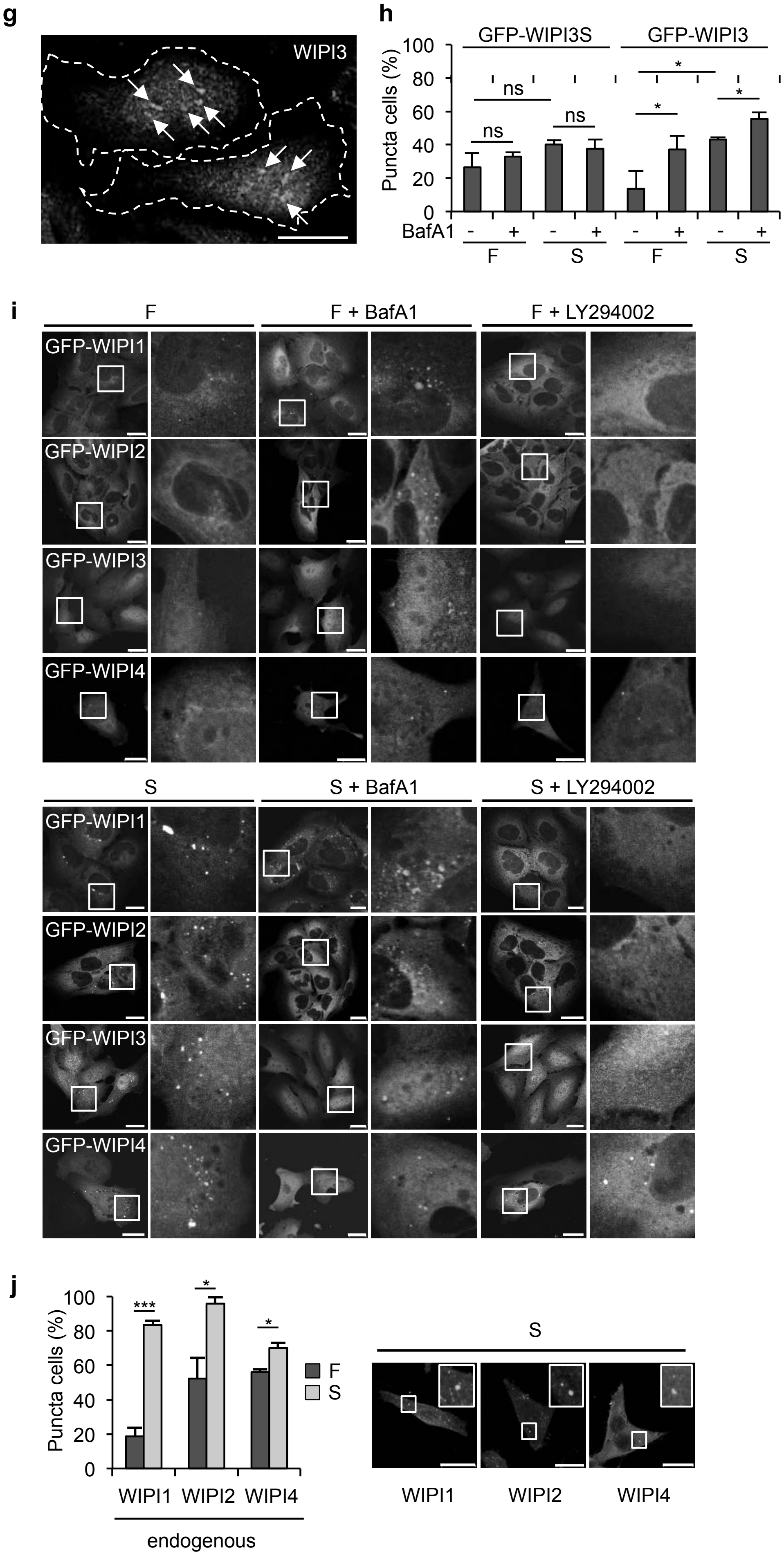

Supplementary Figure 1 (a) Multiple protein sequence alignments of WIPI1, WIPI2B, WIPI3 and WIPI4. Two arginine residues crucial for phospholipid binding are conserved and highlighted with bold red letters in all WIPI sequences. Further amino acids homologous in WIPI proteins and further PROPPIN members[1] are highlighted with red letters in WIPI1 only. Black letters in the WIPI3 protein sequence represent the original sequence (referred to as WIPI3S hereafter), blue letters indicate the new extended WIPI3 N-terminal sequence cloned in this study (GenBank accession number KX434429). (b) Scheme of PIP strip membranes used in this study (left). Phospholipid-protein overlay assays of GFP-WIPI3S or GFP-WIPI3 transiently expressed in U2OS cells (right panels). (c) As indicated, a phospholipid-binding mutant of WIPI3 R230A/R231A was used along with GFP-WIPI3 WT for phospholipid-protein overlay assays (upper panels) and immunoblotting (lower panels). (d) Phospholipid-protein overlay assays of G361 cells immunoblotted with anti-WIPI1, anti-WIPI2 or anti-WIPI4 antibodies (boxed sections presented in Figure 1b). Phospholipid-protein overlay assays of stable GFP-WIPI1, GFP-WIPI2B, GFP-WIPI2D, GFP-WIPI3 U2OS cells (boxed sections presented in Figure 1b) or U2OS cells transiently expressing GFP-WIPI4 with anti-GFP antibodies. (e) Supporting full-cell images for magnified sections (white boxes) in Figure 1b (cell boundaries: dotted lines). (f) Supporting full panels for magnified merged sections (white boxes) in Figure 1d (cell boundaries: dotted lines). (g) Starved G361 cells (3 h) were immunostained for confocal LSM using anti-WIPI3/IgG-Alexa Fluor 488 antibodies (cell boundaries: dotted lines, WIPI3 puncta: arrows). (h) U2OS cells transiently expressing GFP-WIPI3S or GFP-WIPI3 were fed (F) or starved (S) with or without BafA1. The mean percentages of GFP-WIPI3-puncta-positive cells were calculated (up to 336 cells per condition, n=3). (i) Representative images of cells quantified in Figure 1c. (j) G361 cells were fed (F) or starved (S) for 3 h and immunostained using anti-WIPI1/IgG-Alexa Fluor 488, anti-WIPI2/IgG-Alexa Fluor 488 or anti-WIPI4/IgG-Alexa Fluor 488 antibodies. The mean percentages of endogenous WIPI puncta-positive cells (up to 350 cells per condition, n=3) are presented. Mean ± SD; heteroscedastic t-testing; *p*-values: *$p<0.05$; **$p<0.01$; ***$p<0.001$; ns: not significant. Scale bars: 20 μm. Statistics and source data can be found in Supplementary Data 1.

Supplementary Figure 2

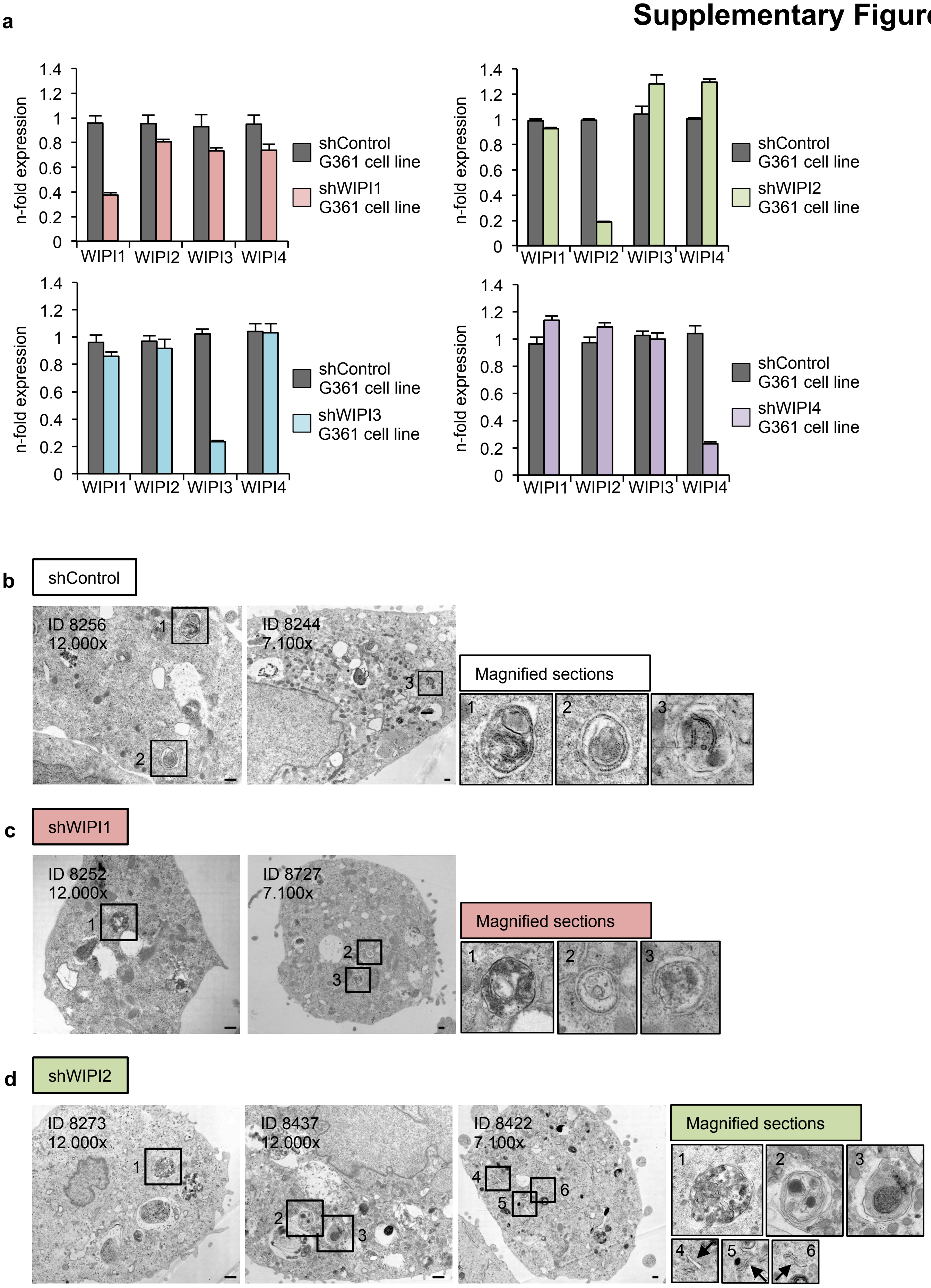

Supplementary Figure 2

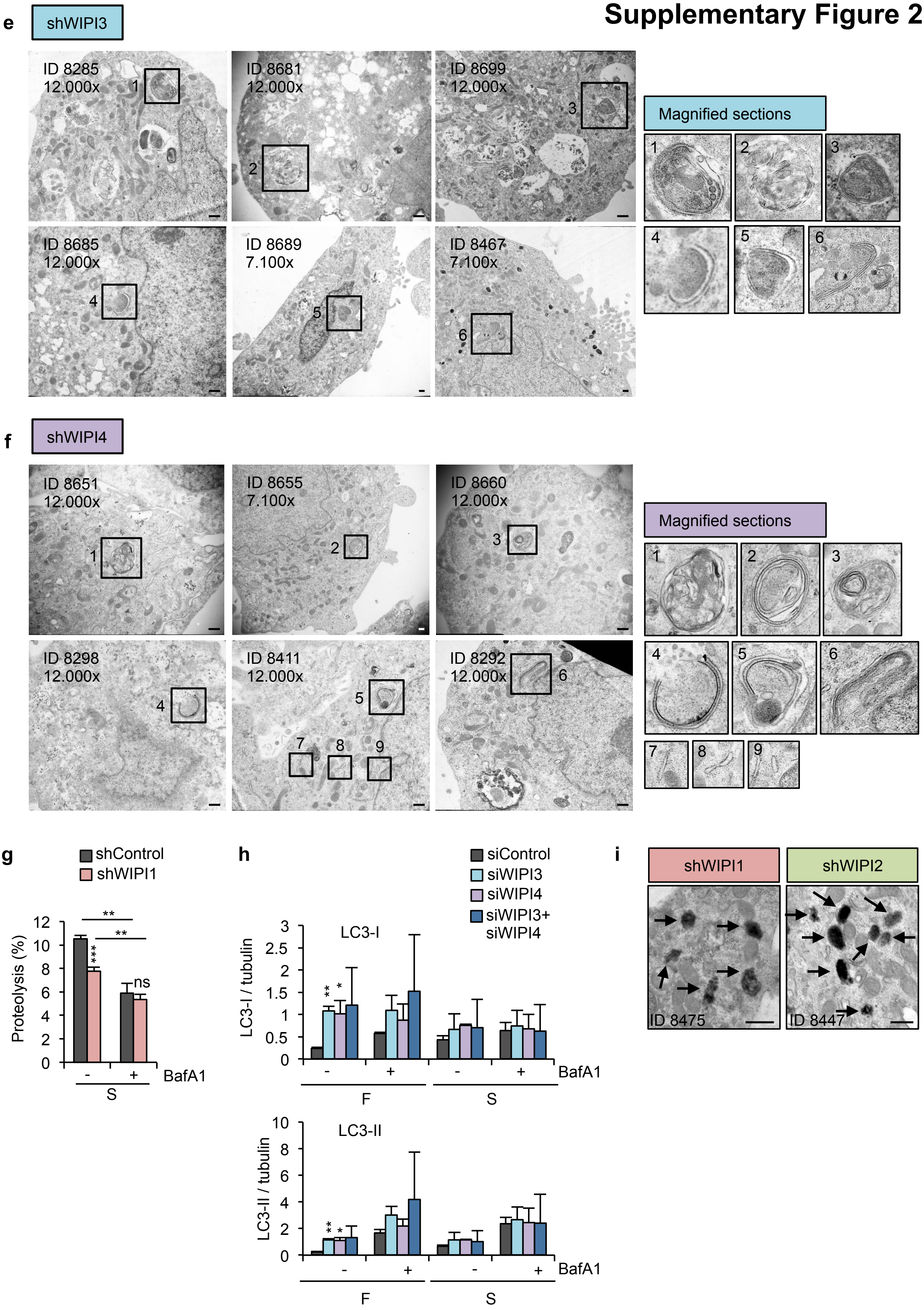

Supplementary Figure 2 (a) Supporting WIPI mRNA abundance assessments by quantitative RT-PCR for results in Figure 2a. (b) Supporting EM images of G361 cells stably expressing control shRNA (shControl). Full EM images: left panels, magnified autophagosomes: right panels). The autophagosome shown in magnified section 1 is presented in Figure 2b. (c) Likewise, supporting EM images of stable G361-WIPI1-KD cells (shWIPI1) are presented. The autophagosome in magnified section 1 is presented in Figure 2b. Autophagosomal structures in G361-WIPI1-KD cells were 2.65 fold reduced when compared to shControl cells. (d) Supporting EM images of stable G361-WIPI2-KD (shWIPI2). Magnified sections (upper panel) indicate the appearance of autophagsomes (reduced by 2.94 fold when compared to shControl cells). RER tubular structures, indicative of an inhibition of early steps in autophagosome formation, increased by 4.93 fold in G361-WIPI2-KD cells (see magnified sections in lower panel, magnified section 4 is presented in Figure 2b). (e) Supporting EM images of stable G361-WIPI3-KD (shWIPI3). The appearance of autophagosomes (magnified sections, upper panel) was reduced by 6.42 fold when compared to shControl cells while additionally, elongated phagophore-like structures appeared (magnified sections, lower panel). Magnified section 6 is presented in Figure 2b. (f) Supporting EM images of stable G361-WIPI4-KD cells. Autophagosomal structures (magnified sections, upper panel) were reduced by 3.47 fold when compared to shControl cells. Also, elongated phagophore-like structures appeared (magnified sections, middle panel, section 4 is presented in Figure 2b). Moreover, RER tubular structures (magnified sections, lower panel) accumulated by 3.91 fold when compared to shControl cells. (g) Stable G361-WIPI1-KD (shWIPI1) and control (shControl) cells were starved (S) for 3 h in the absence or presence of BafA1 and the percentages of autophagic proteolysis of long-lived proteins upon starvation (with or without bafilomycin A1, BafA1) were calculated based on triplicate sets (n=1). (h) Full quantification of western blot results shown in Figure 2f. (i) Appearance of melanosomes (arrows) in G361-WIPI1-KD (shWIPI1) and G361-WIPI2-KD (shWIPI2) cells. Scale bars: 500 nm. Mean ± SD; heteroscedastic t-testing; *p*-values: *$p<0.05$; **$p<0.01$; ***$p<0.001$; ns: not significant. EM magnifications are indicated (12.000x or 7.100x). Statistics and source data can be found in Supplementary Data 1.

Supplementary Figure 3

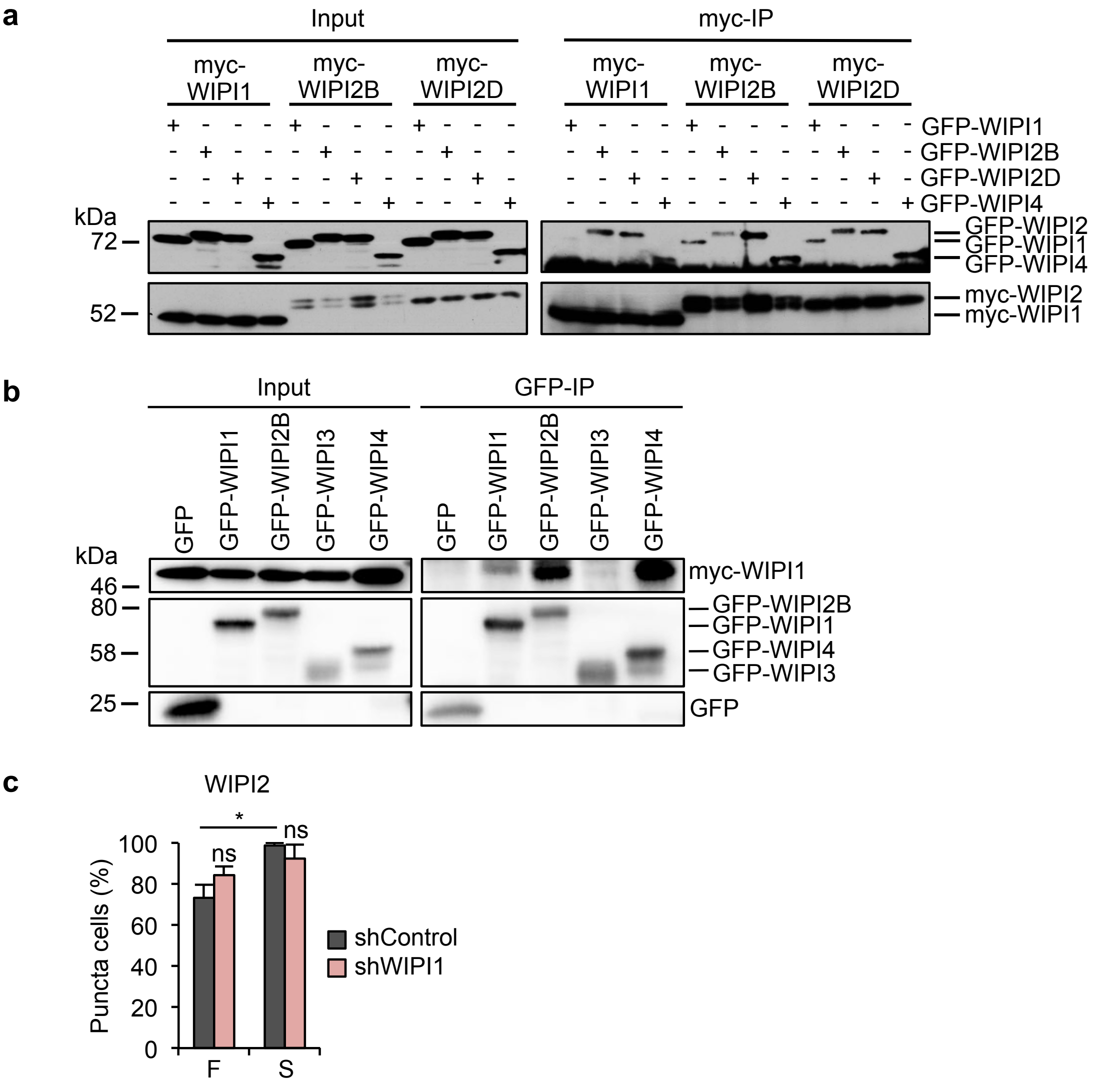

Supplementary Figure 3 (a) U2OS cells transiently expressing GFP- (WIPI1, WIPI2B, WIPI2D, WIPI4) or myc-tagged WIPI proteins (WIPI1, WIPI2B, WIPI2D) were starved for 3 h, analysed by immunoprecipitation using anti-myc antibody and immunoblotted with anti-GFP or anti-myc antibodies. Input samples are shown on the left, immunoprecipitates (myc-IP) on the right. Associations between WIPI1, WIPI2B and WIPI4 are demonstrated. (b) Self-association of WIPI1, and association between WIPI1 and WIPI2B, as well as between WIPI1 and WIPI4 was confirmed by analysing U2OS cells transiently co-expressing GFP-WIPI1, GFP-WIPI2B or GFP-WIPI4 and myc-tagged WIPI1. Immunoprecipitation of GFP-WIPI3 was negative for myc-tagged WIPI1. (c) Stable G361-WIPI1-KD (shWIPI1) and control (shControl) cells were fed (F) or starved (S) for 3 h and endogenous WIPI2 immunostained with anti-WIPI2/IgG-Alexa Fluor 488 antibodies for confocal LSM. The mean percentages of WIPI2 puncta-positive cells were calculated (up to 114 cells per condition, n=3). Mean ± SD; heteroscedastic t-testing; *p*-values: *$p<0.05$; ns: not significant. Statistics and source data can be found in Supplementary Data 1.

Supplementary Figure 4

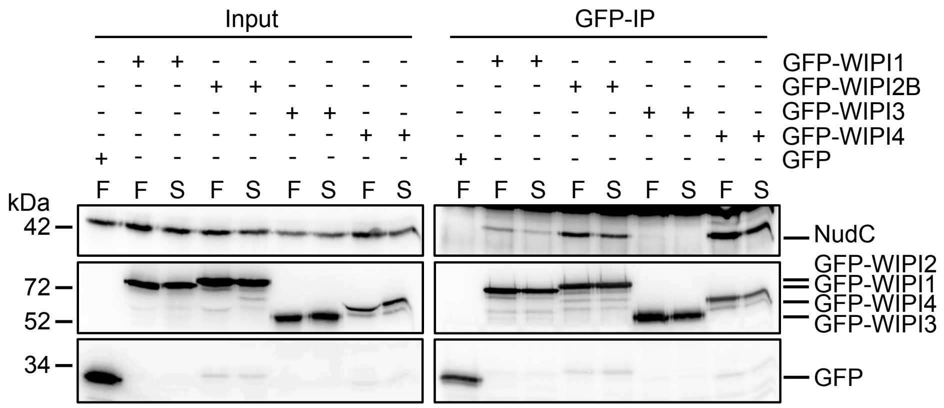

Supplementary Figure 4 U2OS cells stably expressing GFP-WIPI1, GFP-WIPI2B, GFP-WIPI3, GFP-WIPI4 or GFP were fed (F) or starved (S) for 3 h and immunoprecipitated using anti-GFP antibodies. Immunoblotting was performed using anti-GFP and anti-NudC antibodies.

Supplementary Figure 5

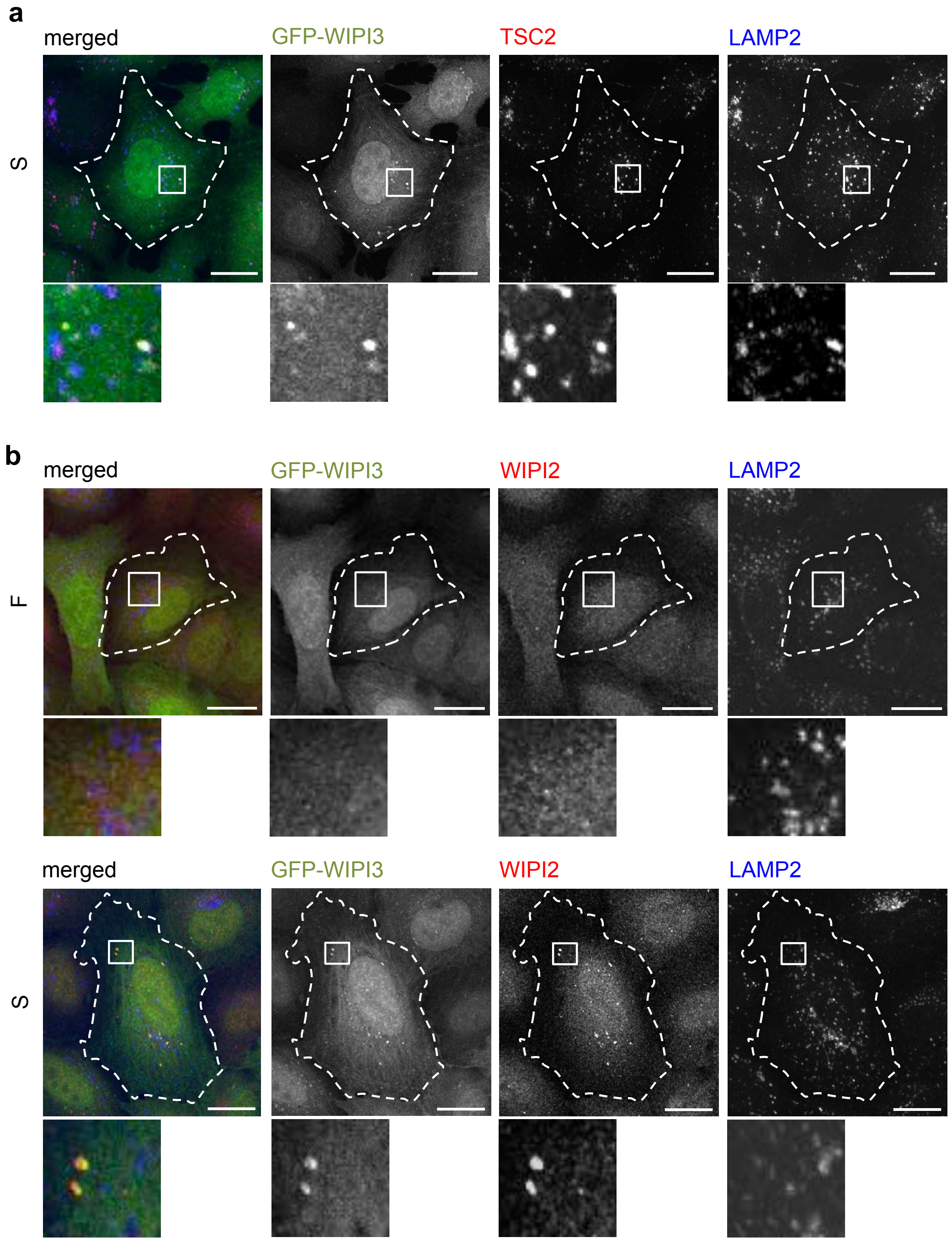

Supplementary Figure 5

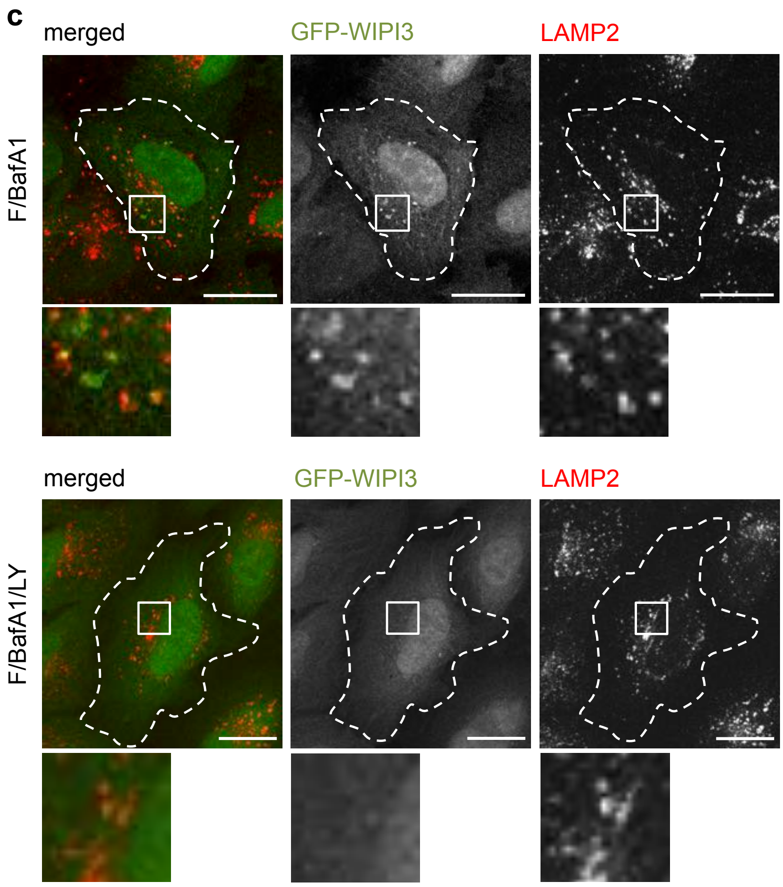

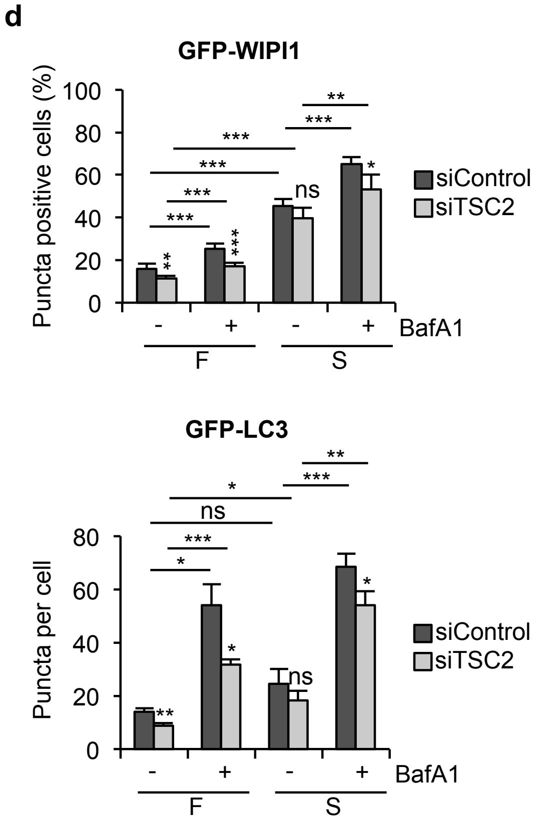

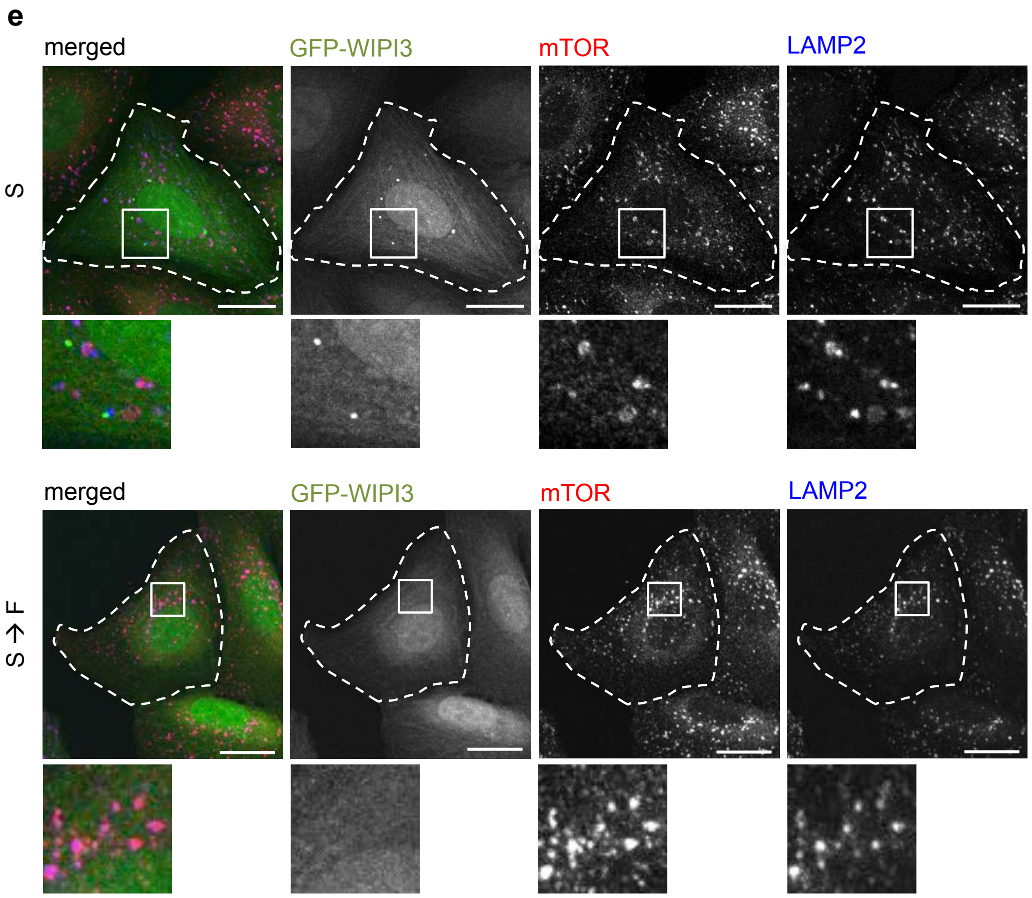

Supplementary Figure 5 (a) Cell images of the magnified sections (shown here in the lower panels again) represented in Figure 5g. (b) Cell images of the magnified sections (shown here in the lower panels again) represented in Figure 5h. (c) U2OS cells stably expressing GFP-WIPI3 were cultured for 3 h in control medium (F) supplemented with BafA1 in the absence (upper panel) or presence (lower panel) of LY294002 (LY). Cells were immunostained with anti-LAMP2/IgG-Alexa Fluor 546 antibodies and visualised by confocal LSM. Cell images and magnifications (white boxes) are presented. (d) U2OS cells stably overexpressing GFP-WIPI1 or GFP-LC3 and transiently transfected with control siRNA or TSC2-targeting siRNA were fed (F) or starved (S) in the absence or presence BafA1. Assessments of GFP-WIPI1 (upper panel) and GFP-LC3 puncta (lower panel) were conducted using an In Cell Analyzer 1000. Images from up to 7,771 GFP-WIPI1 (n=5) or up to 4,697 GFP-LC3 cells per condition (n=3) were analysed. Mean numbers (+/-SD) of GFP-WIPI1-puncta-positive cells (%) or GFP-LC3 puncta per cell are presented. (e) Cell images of magnified sections in Figure 5k (shown here in the lower panels again) are presented. Cell boundaries are indicated (dotted lines). Scale bars: 20 μm. P-values: *$p<0.05$; **$p<0.01$; ***$p<0.001$; ns: not significant. Statistics and source data can be found in Supplementary Data 1.

Supplementary Figure 6

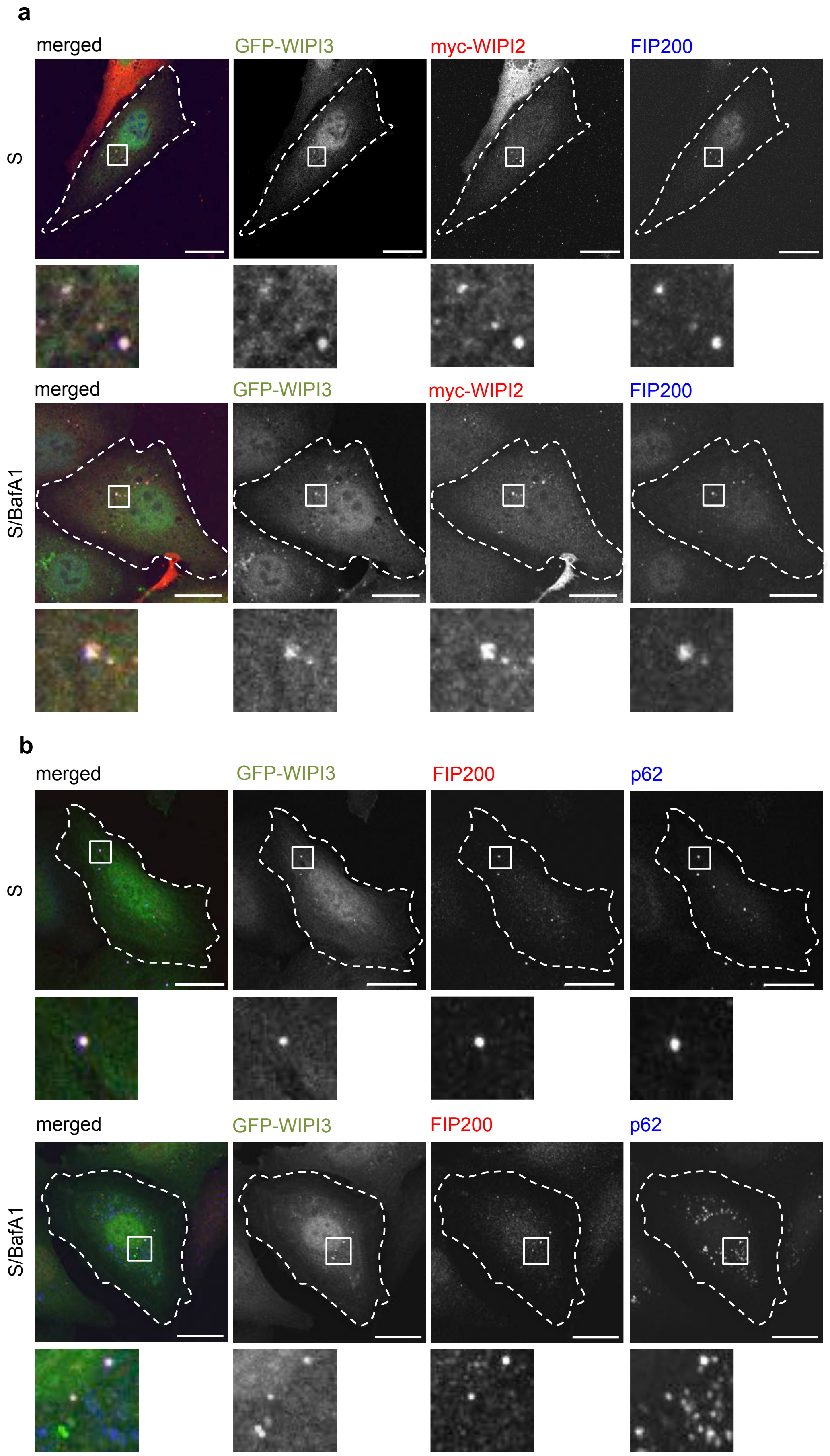

Supplementary Figure 6

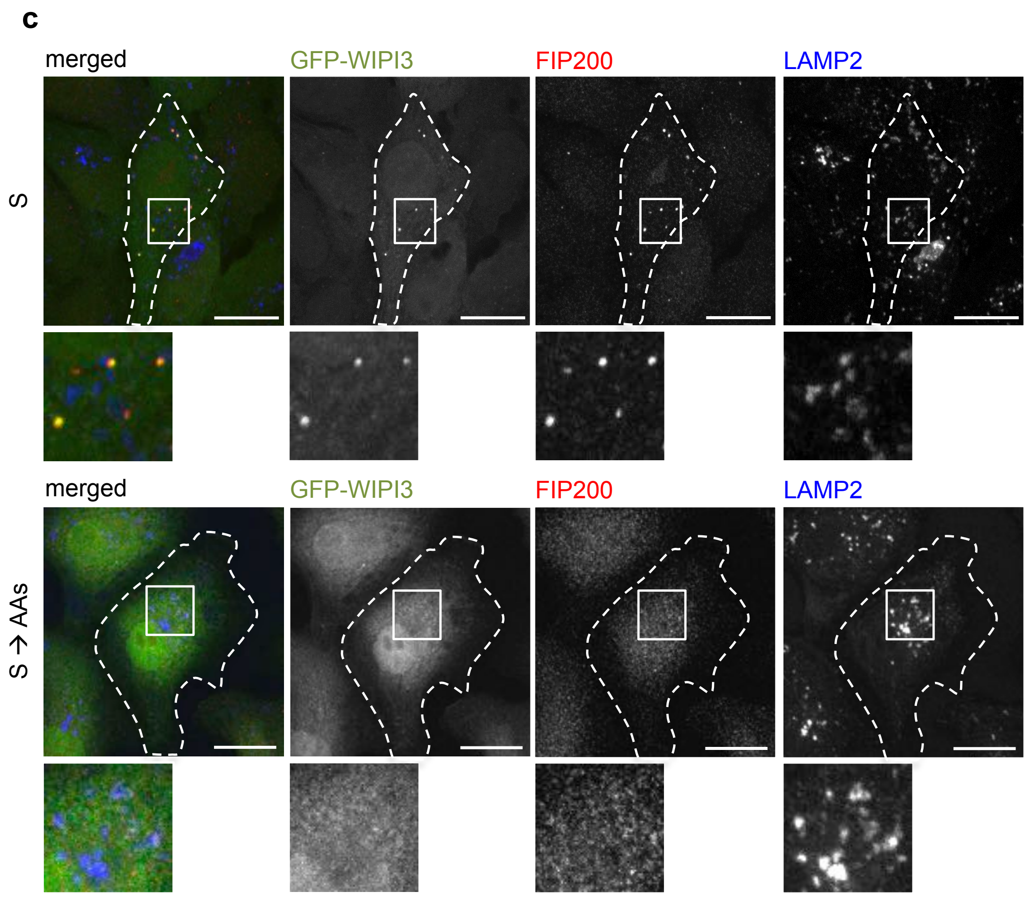

Supplementary Figure 6 (a) Cell images of magnified sections (white boxes) in Figure 6b (shown here in the lower panels again) are presented. (b) Cell images of magnified sections (white boxes) in Figure 6c (shown here in the lower panels again) are presented. (c) Cell images of magnified sections (white boxes) in Figure 6h (shown here in the lower panels again) are presented. Cell boundaries are indicated (dotted lines). Scale bars: 20 μm.

Supplementary Figure 7

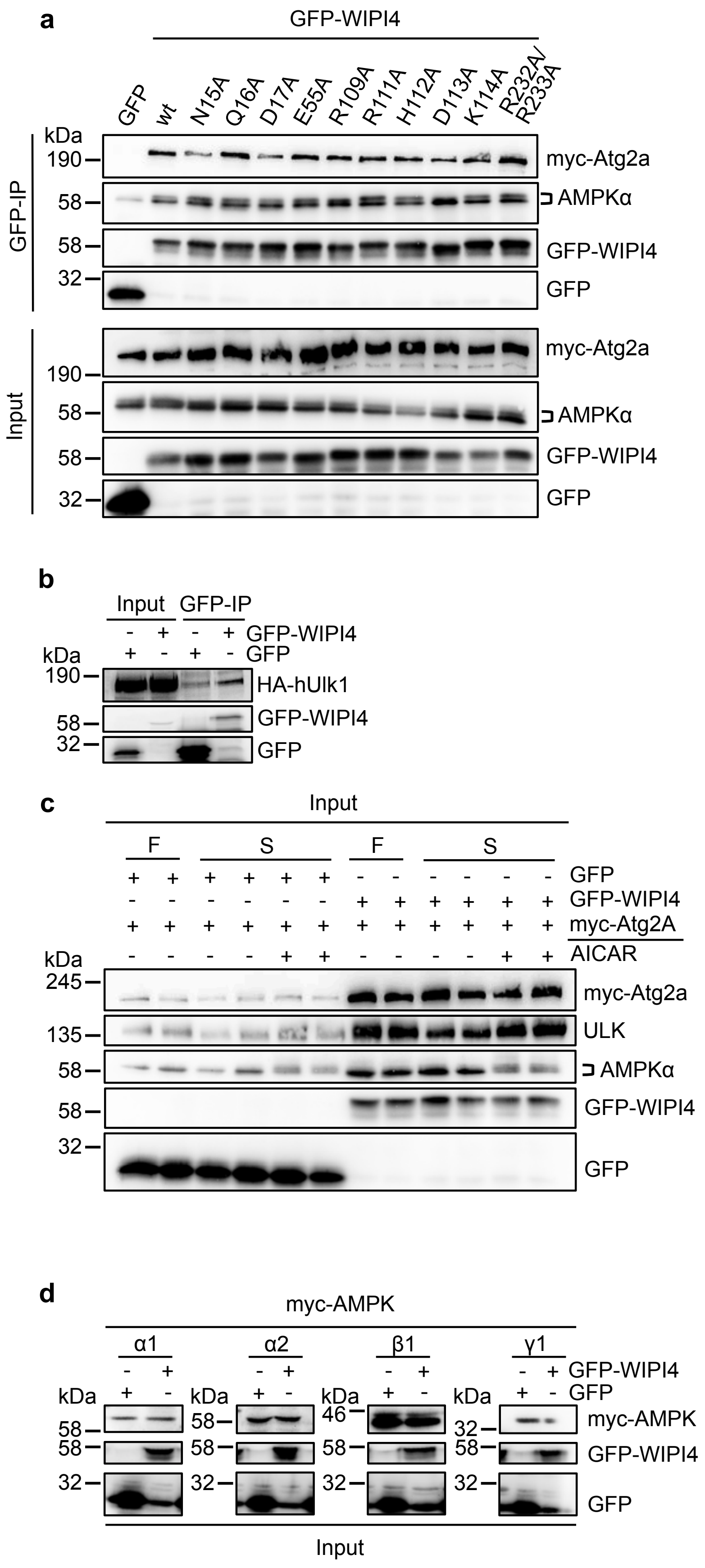

Supplementary Figure 7 (a) Cell lysates of U2OS cells transiently expressing GFP or GFP-tagged WIPI4 wild-type (WT) or mutants (amino acid substitutions are indicated for each of the mutant WIPI4 proteins) and myc-ATG2A were analysed by immunoprecipitation using anti-GFP antibodies (upper panels). Immunoblotting was performed using anti-myc, anti-GFP or anti-AMPKα antibodies. Input samples are indicated (lower panels). (b) Cell lysates of U2OS cells transiently expressing GFP or GFP-WIPI4 and HA-tagged human ULK1 were analysed by immunoprecipitation using anti-GFP antibodies. Immunoblotting was performed using anti-HA and anti-GFP antibodies. (c) Input samples for the results presented in Figure 7h are shown. (d) Input samples for the results presented in Figure 7k are presented.

Supplementary Figure 8

a

Lentiviral-based shRNA screen targeting the human kinome for autophagy assessments

Primary Screen

Lentiviral-based shRNA library with 3109 different shRNAs targeting 673 human protein kinases

↓

Infection of U2OS cells stably expressing GFP-WIPI1 in 96-well plates

↓

Serum starvation (3 h)

↓

Automated high through-put GFP-WIPI1 puncta image acquisition and analysis

↓

Selection of candidates based on histogram distribution differences with regard to control infections

Secondary Screen

Generation of monoclonal cell lines stably expressing lentiviral-derived shRNA constructs targeting candidate protein kinases

↓

Amino acid starvation (3 h)

↓

Automated high through-put GFP-WIPI1 puncta image acquisition and analysis

b

Targeted gene	p value
ALPK3	0.027804016
BRD3	0.005053786
BRD4	0.001101193
BRSK2	0.016222751
CAMKK1	0.008694701
CDC2L6	0.012722347
CDC42BPB	0.001565819
CDKL3	0.112191334
CHEK-1	0.547902872
CHUK	0.006735652
CRKRS	0.085376527
DAPK2	0.610800594
DCLK2	0.276489172
DYRK2	0.310518804
EPHA1	0.045141
FRK	0.029239985
HCK	0.161027472
JAK3	0.141298471
LTK	4.95955E-09
MAP3K2	0.62973672
MAP4K1	3.02827E-09
MAP4K4	0.000696531
MAP4K5	0.029414705
MERTK	1.72517E-09
NEK5	6.75408E-08
NTRK3	0.003184975
NUAK2	0.043787497
OXSR1	0.002543365
PDGFRB	4.86401E-05
PDIK1L	0.002170912
PDK2	0.90075686
PDK3	0.041611488
PFTK1	0.26359669
PLK2	0.546526909
PRKAG1	0.283281129
PRKCD	0.106740127
PRKX	0.390784533
RYK	6.86983E-05
SRMS	4.06524E-05
STK16	0.045557941
STK25	0.481308667
STK33	0.167445238
TRPM7	9.1742E-08
TSSK4	0.000334938
TWF2	0.479403382
TYK2	0.178102335
VRK2	0.208043918
WNK1	0.964108064

Supplementary Figure 8

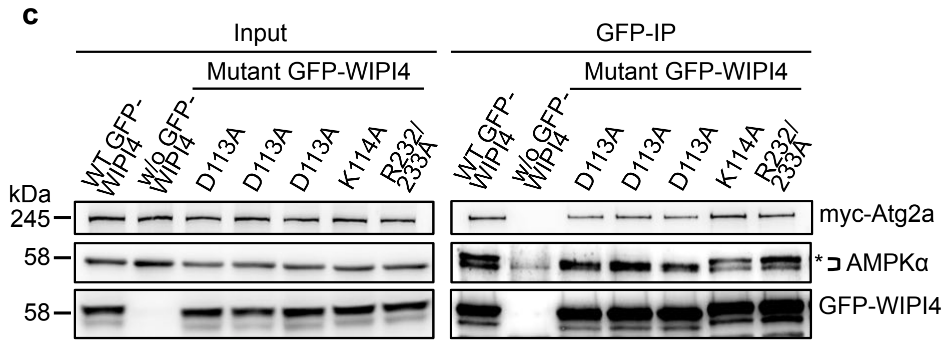

Supplementary Figure 8 (a) Lentiviral-based shRNA screening procedure and results (b) using MISSION LentiExpress Human Kinases (Sigma-Aldrich). See Methods section for details. (c) To identify amino acids in WIPI4 conferring AMPK α binding, a panel of mutant WIPI4 variants was assessed for specific binding to AMPK α (Supplementary Figure 7a). Mutant WIPI4 D113A was identified and its impaired binding to AMPK α (upper band, indicated with an asterisk, is missing) was confirmed (triplicate samples) by anti-GFP immunoprecipitation and immunoblotting with anti-myc, anti-AMPK α and anti-GFP antibodies using cell lysates of U2OS cells transiently transfected with constructs encoding myc-ATG2A and GFP-WIPI4 wild-type (wt) or mutant proteins (D113A, K114A, R232/233A).

Supplementary Figure 9

Figure 1b

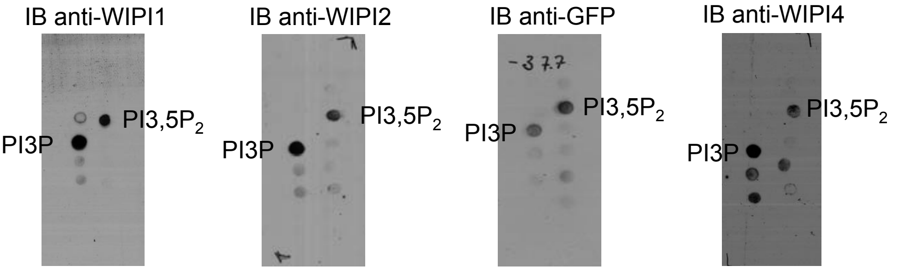

Figure 2f

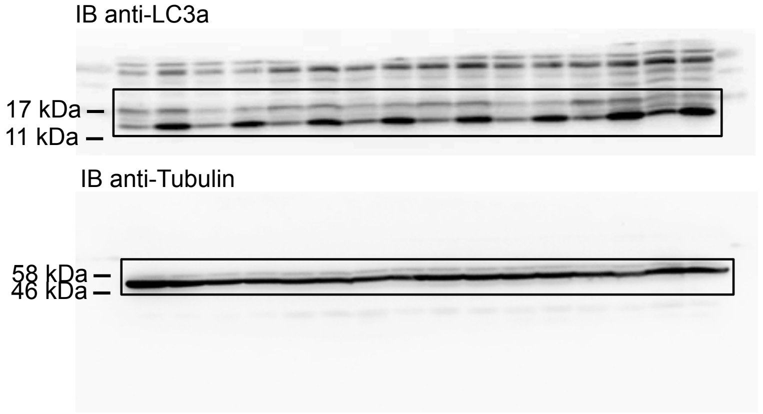

Figure 4a

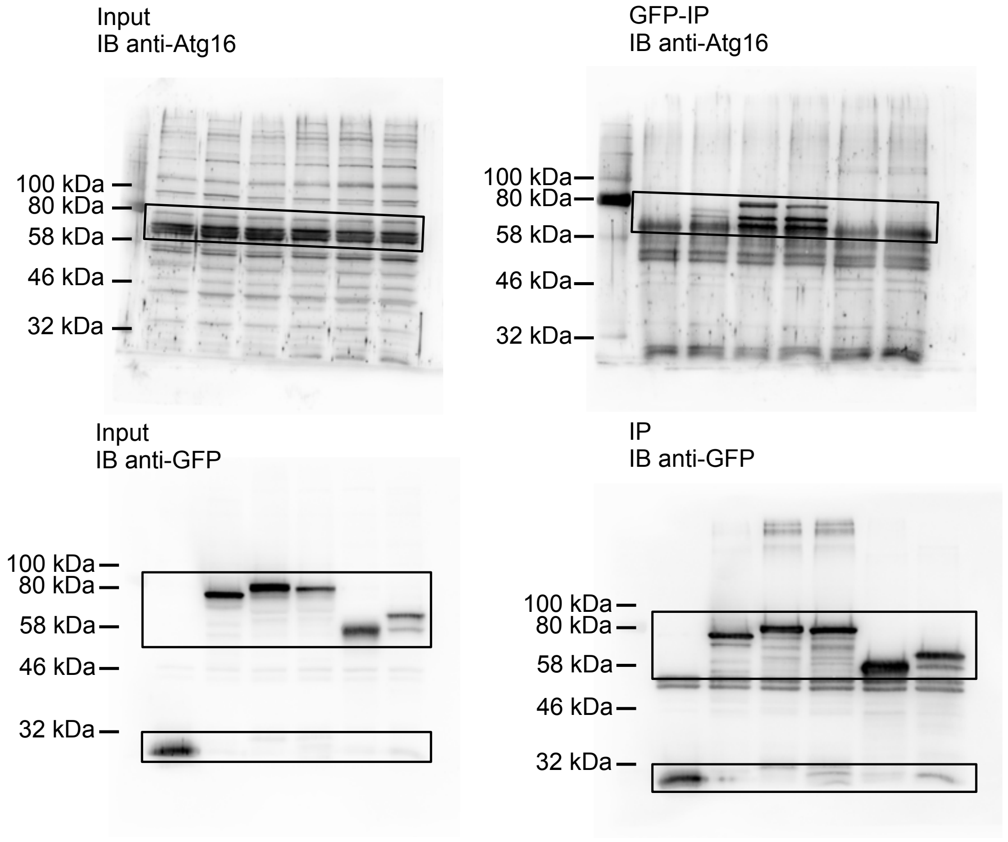

Supplementary Figure 9

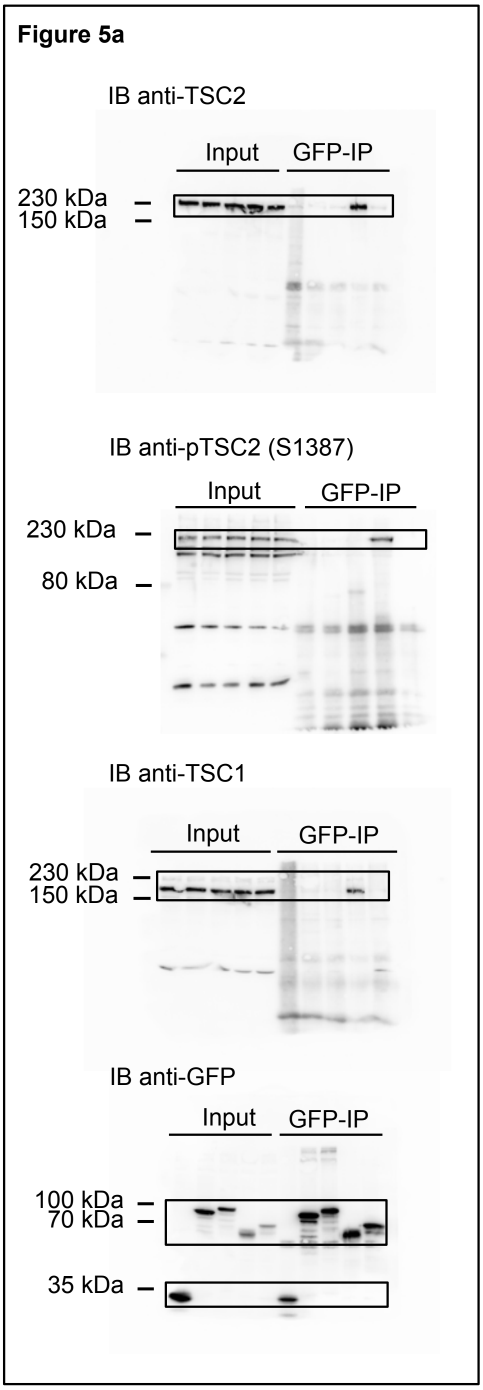

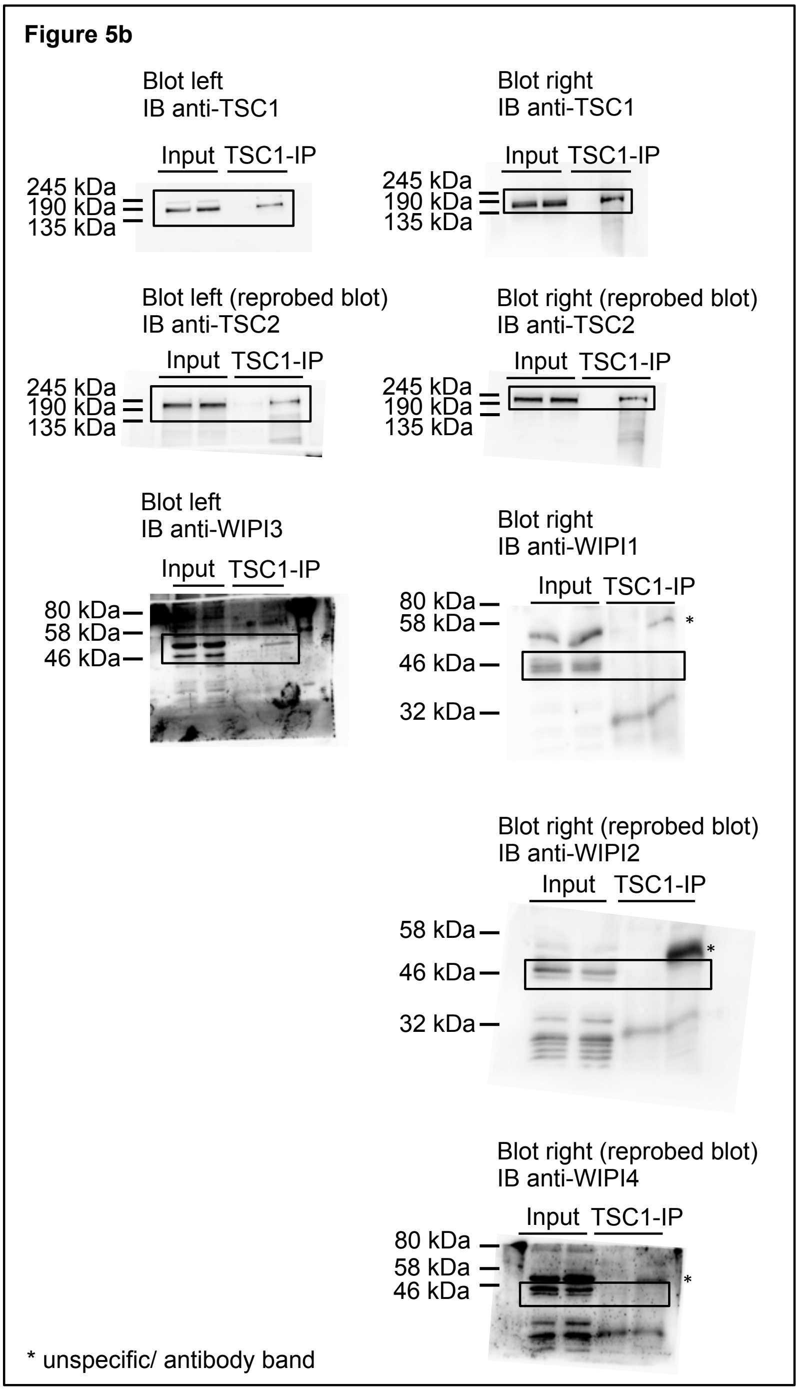

Supplementary Figure 9

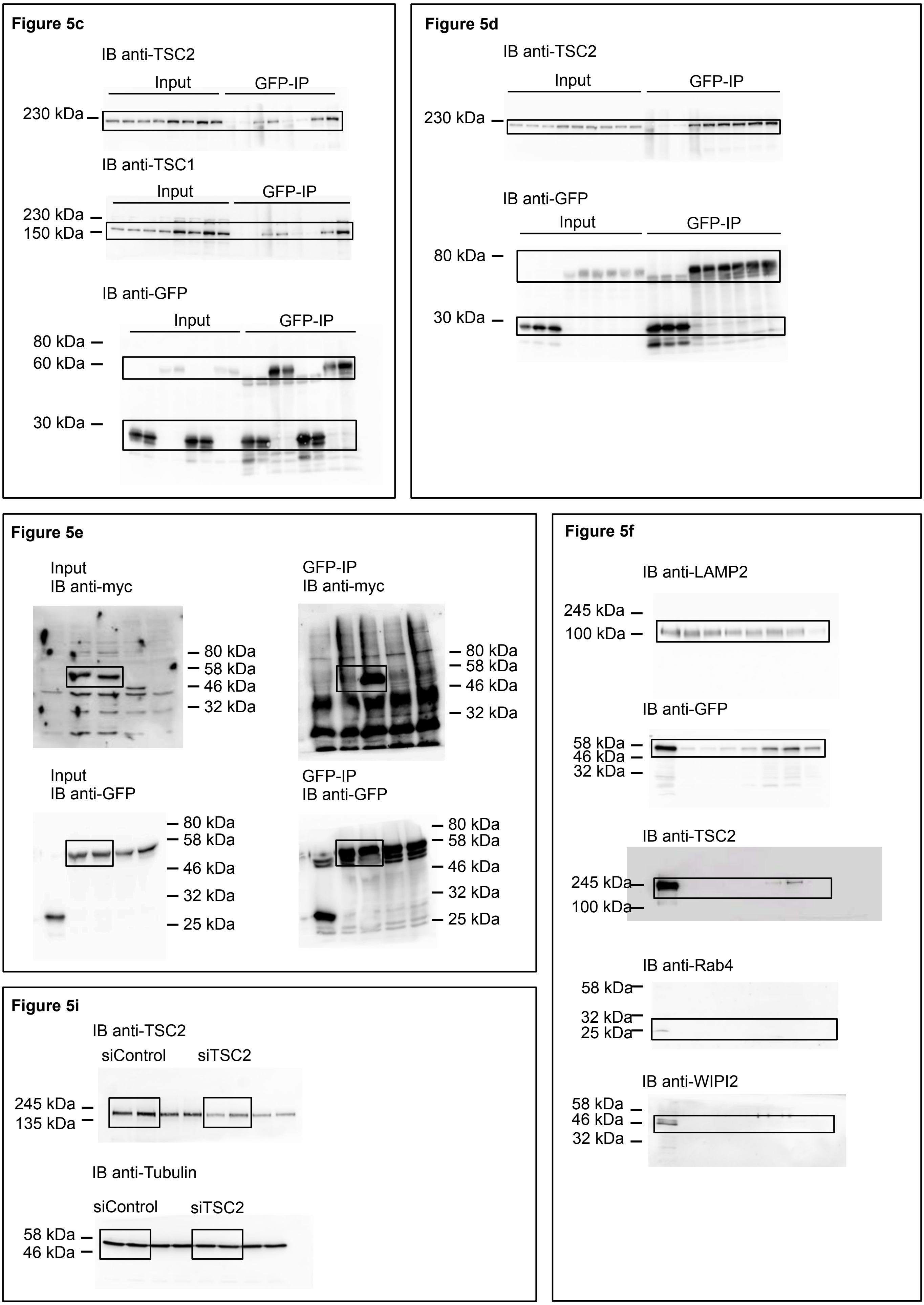

Supplementary Figure 9

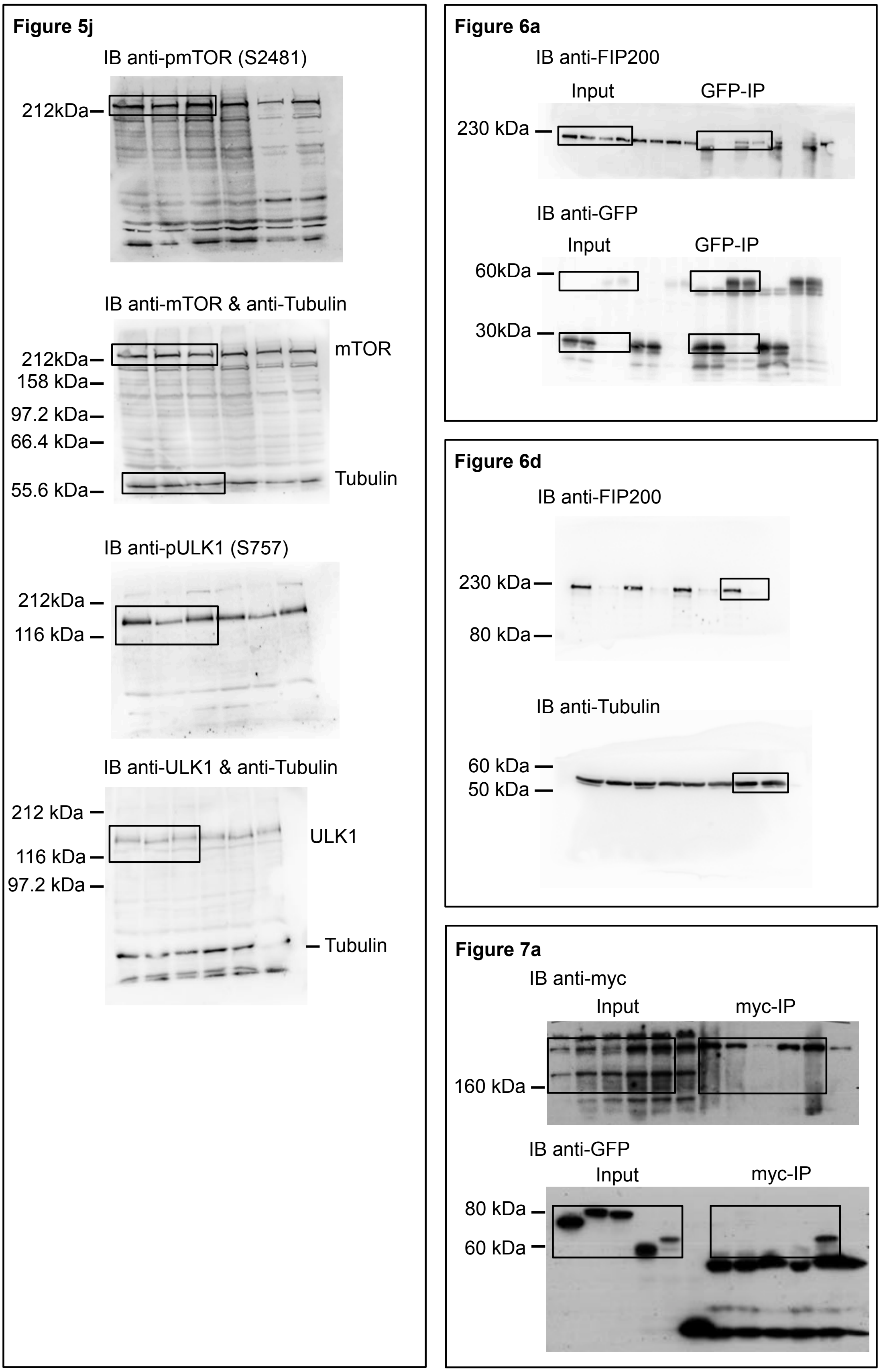

Supplementary Figure 9

Figure 7b

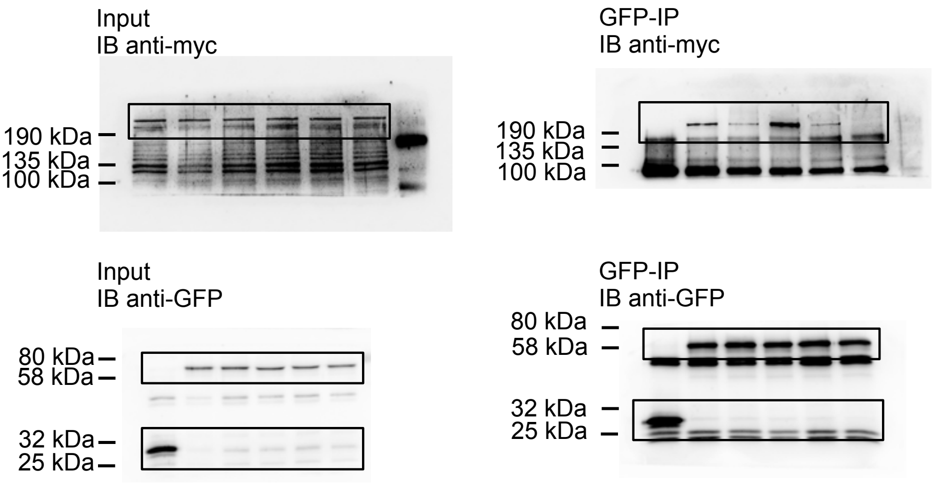

Figure 7f

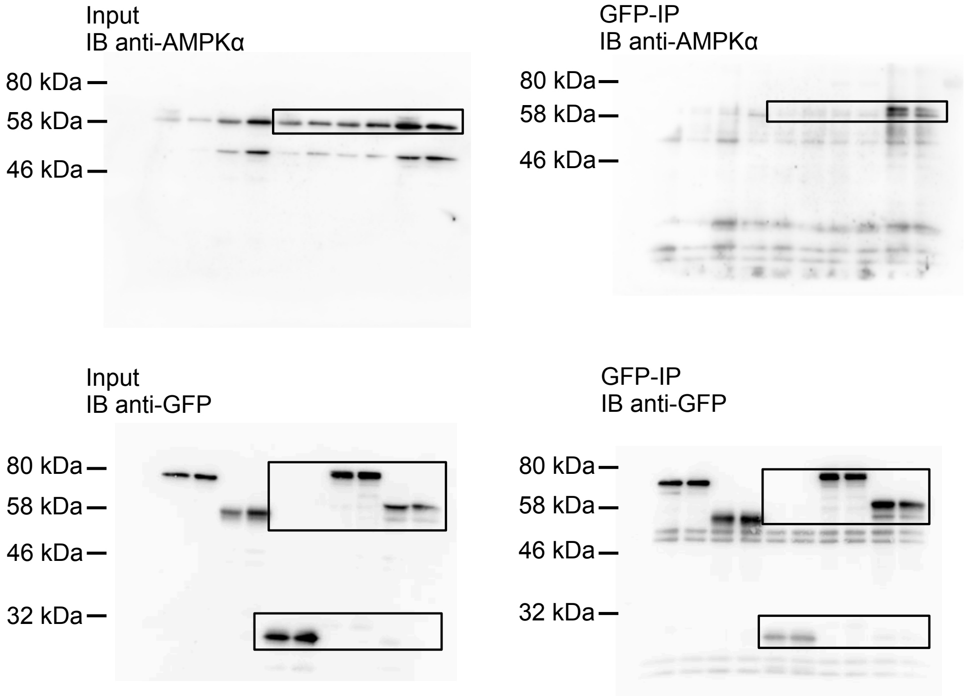

Supplementary Figure 9

Figure 7g

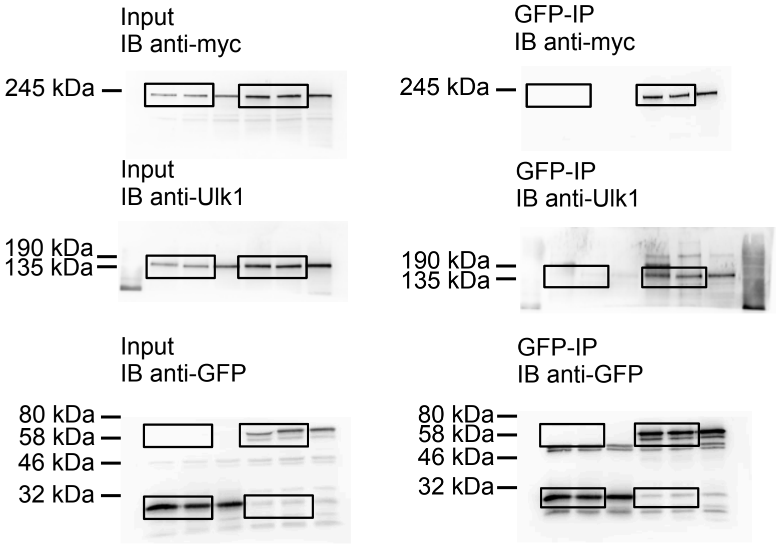

Figure 7h

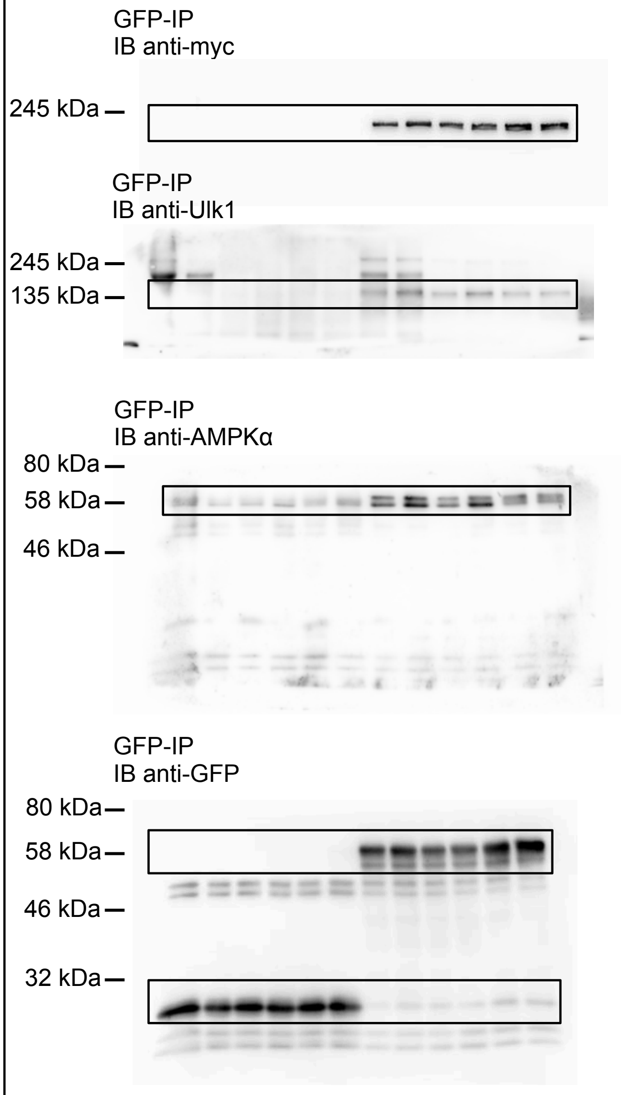

Figure 7j

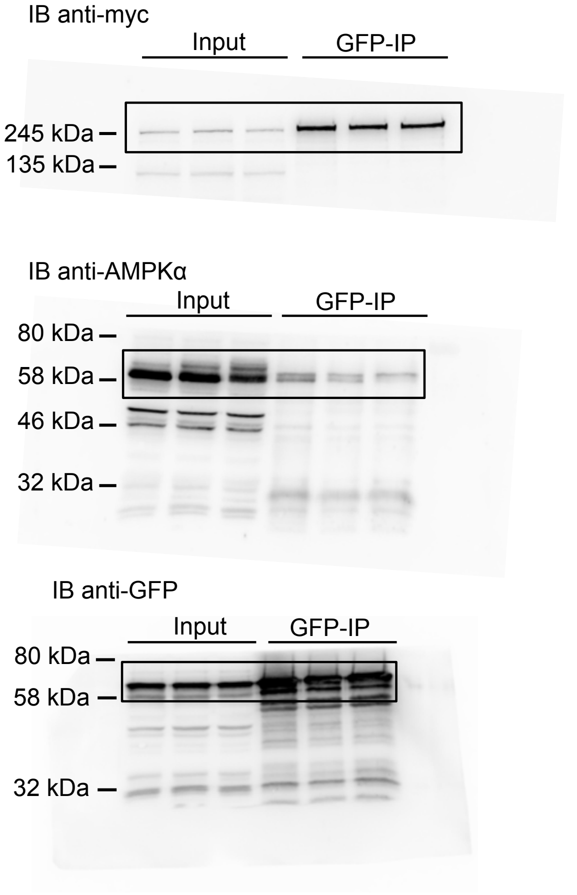

Supplementary Figure 9

Figure 7k

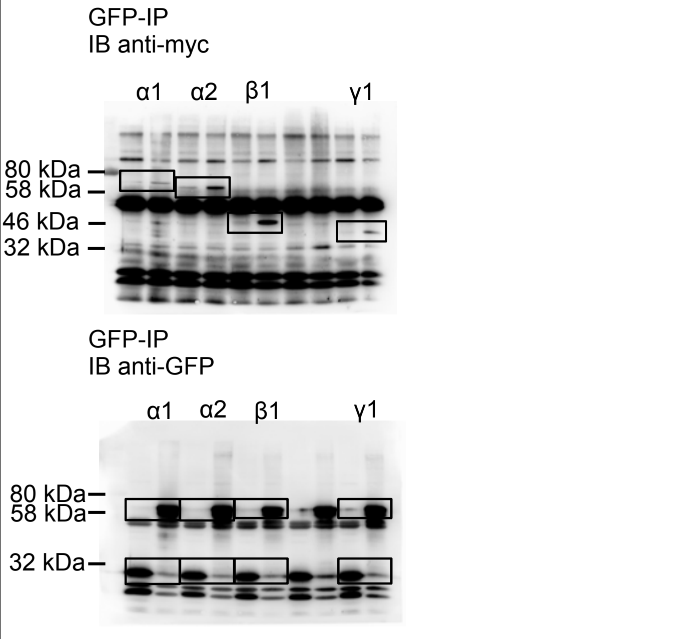

Figure 8d

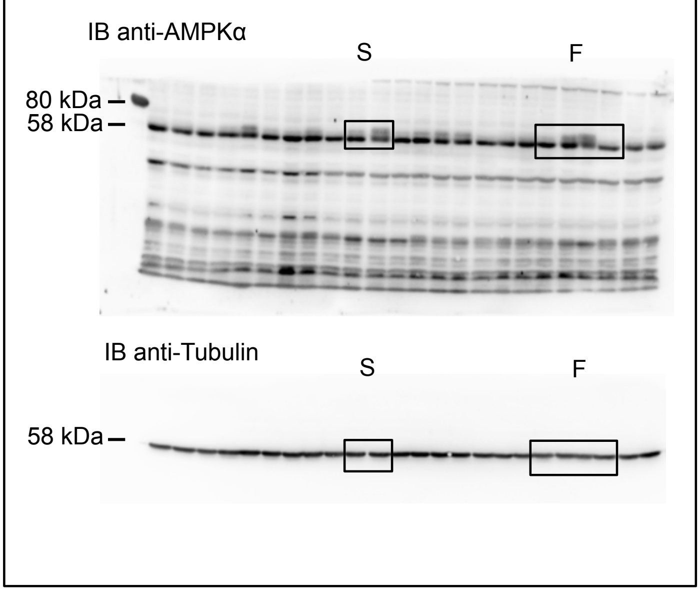

Figure 8b

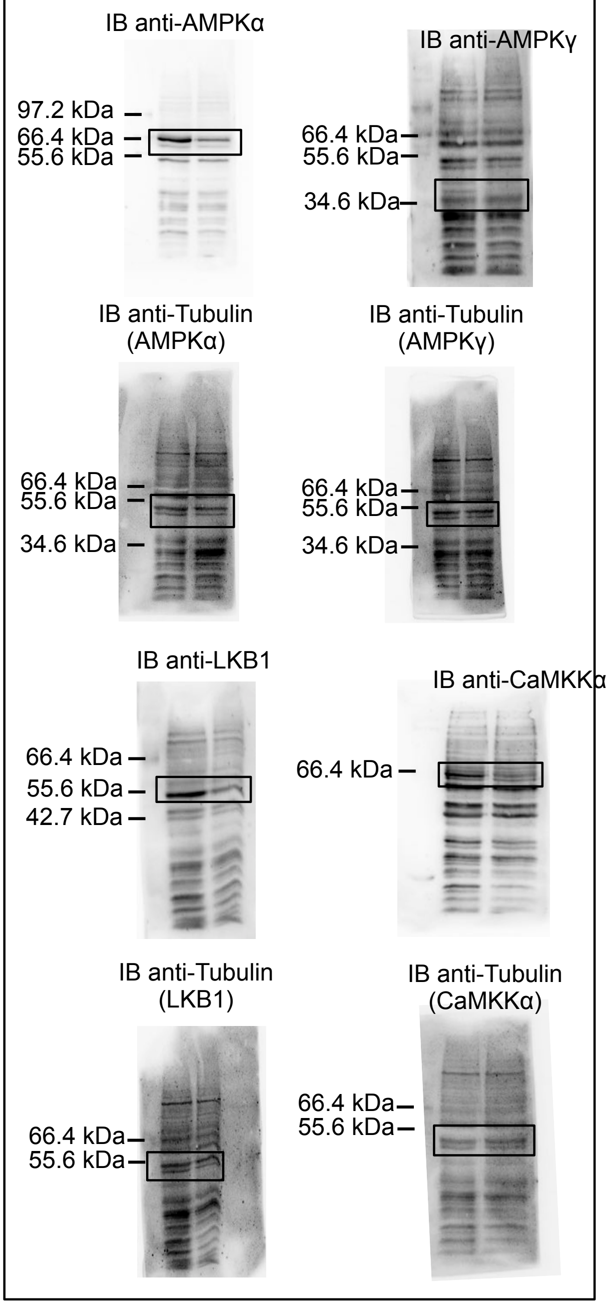

Supplementary Figure 9

Supplementary Figure 1b

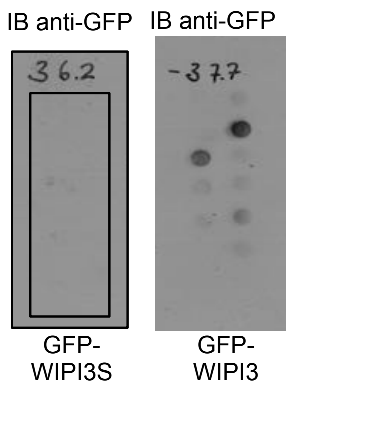

Supplementary Figure 1d

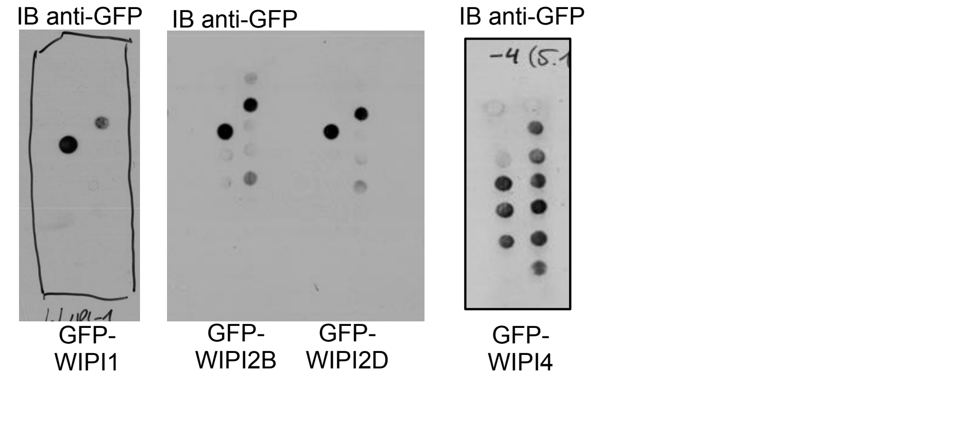

Supplementary Figure 1c

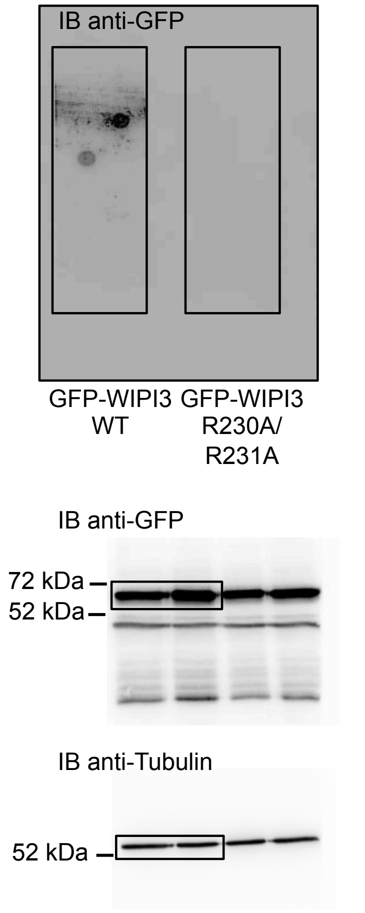

Supplementary Figure 3a

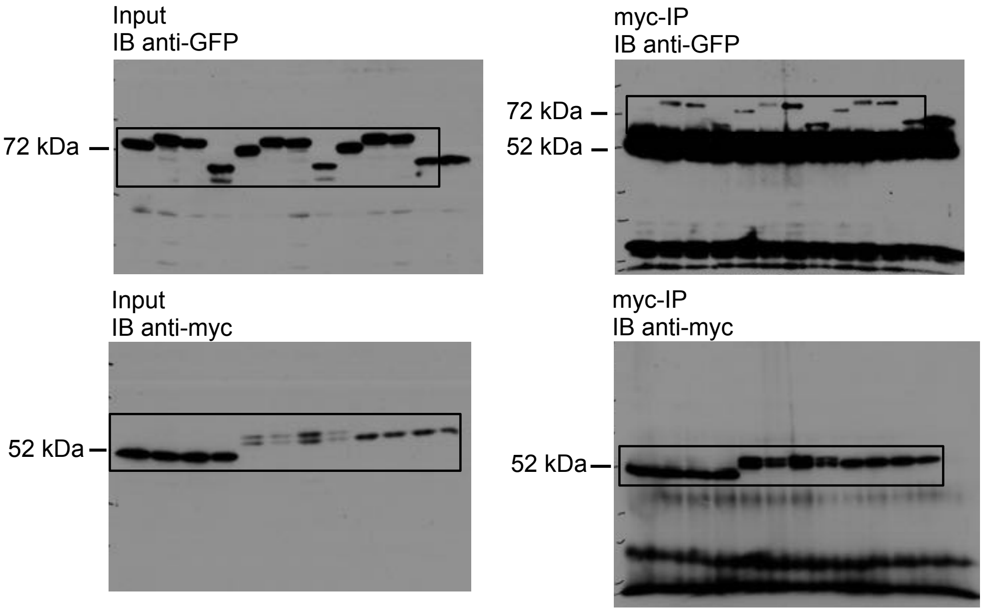

Supplementary Figure 9

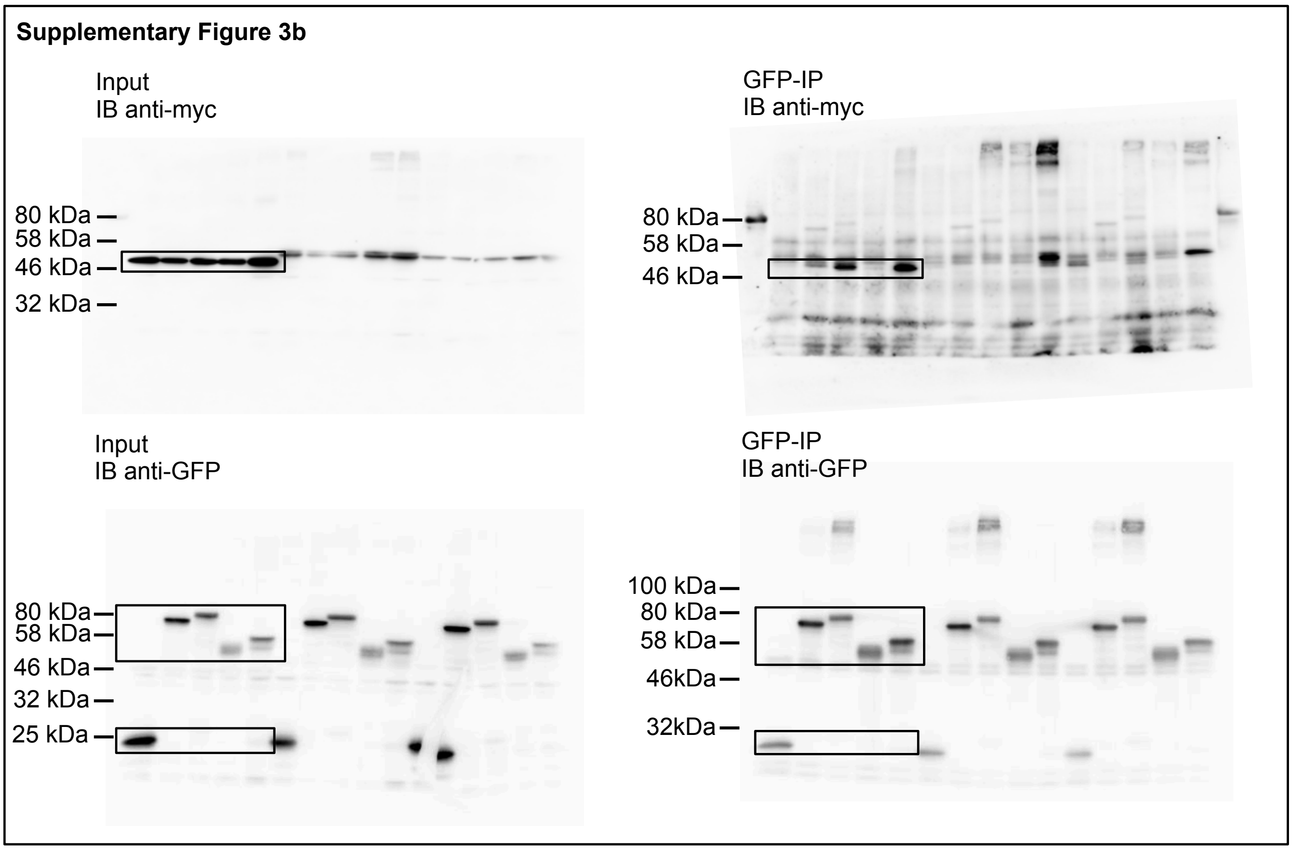

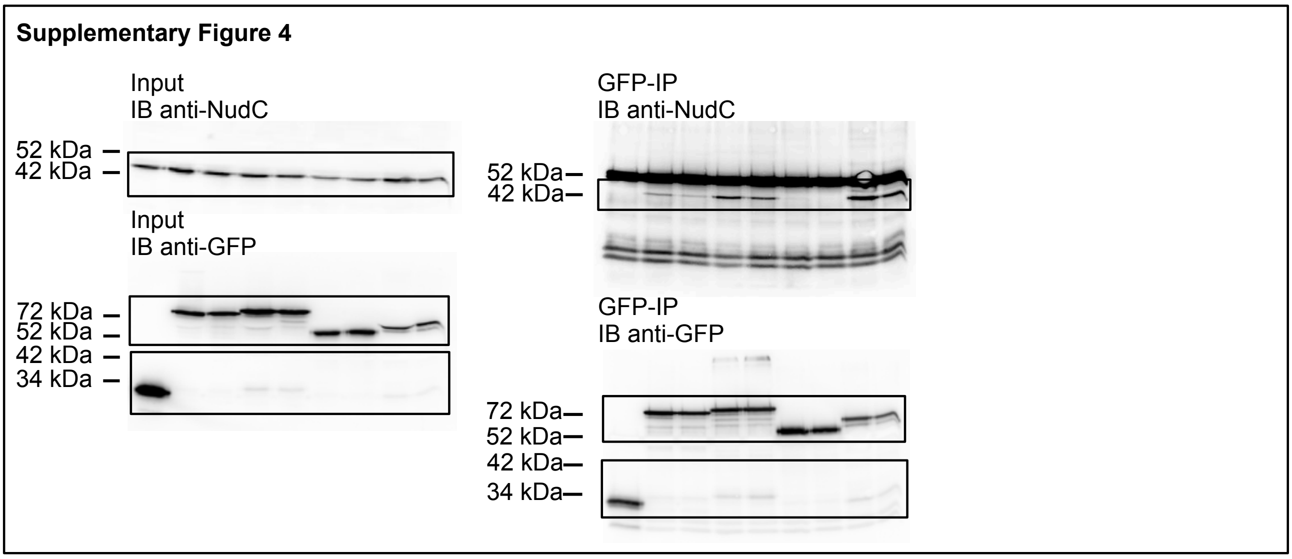

Supplementary Figure 9

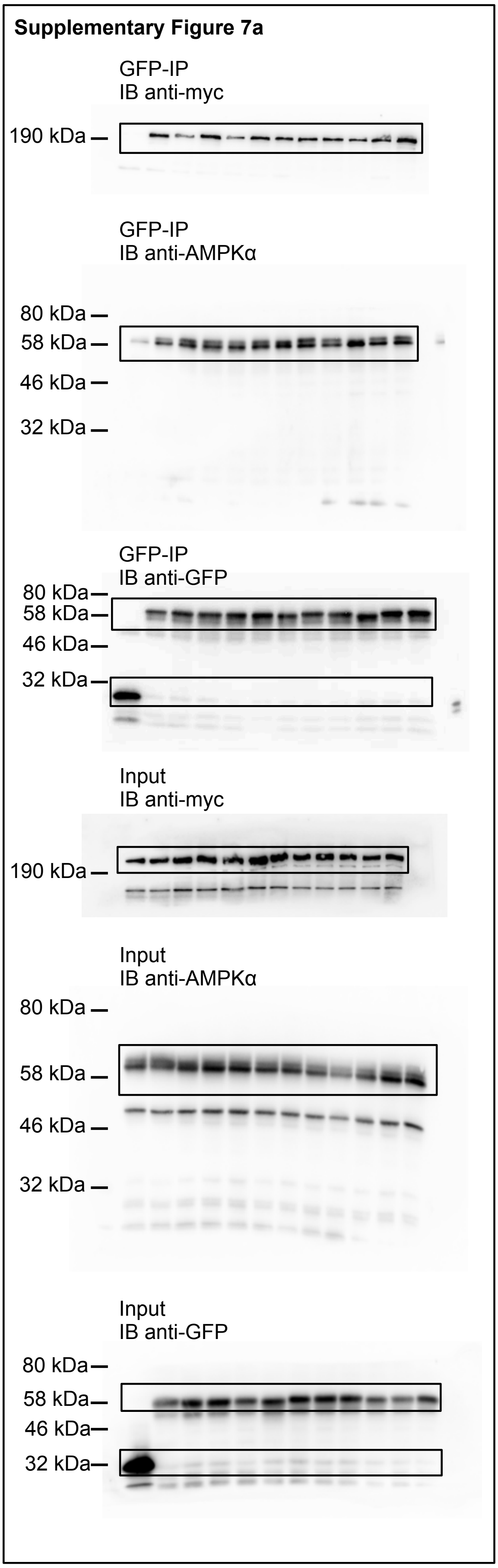

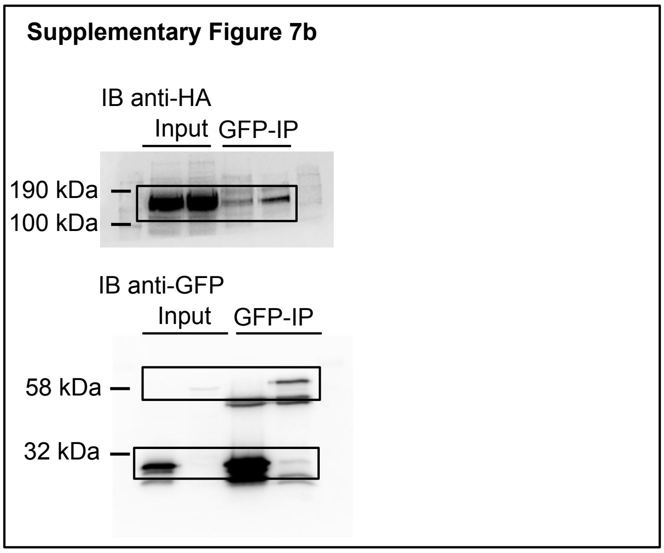

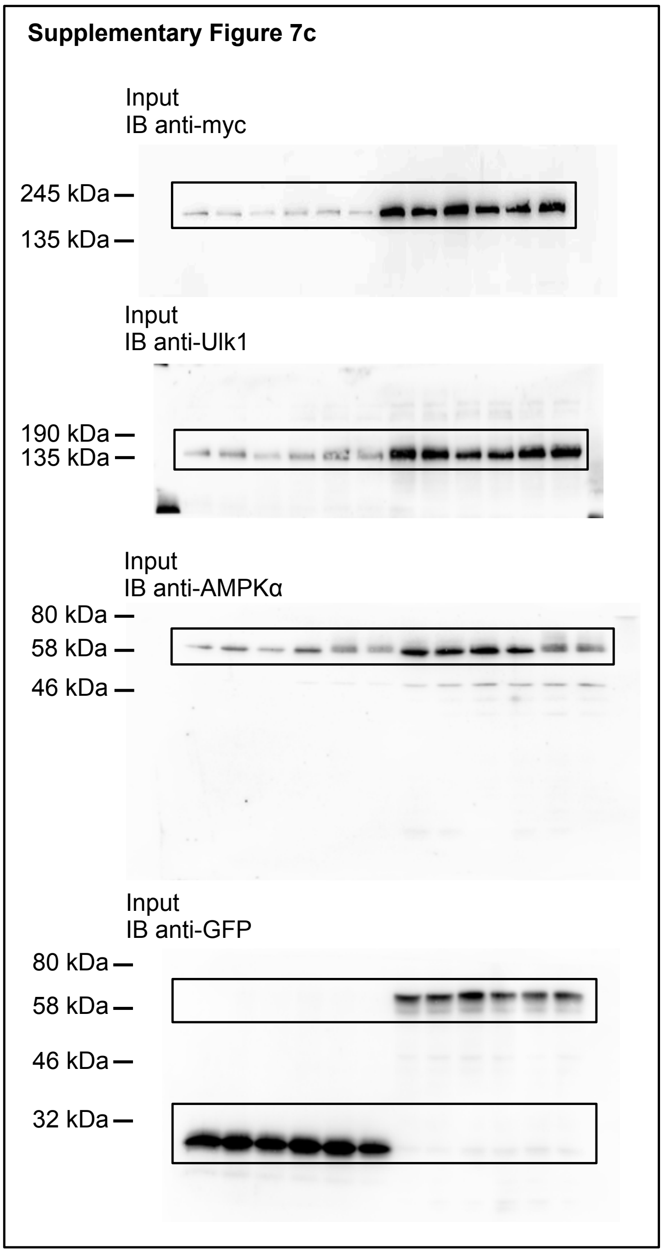

Supplementary Figure 9

Supplementary Figure 7d

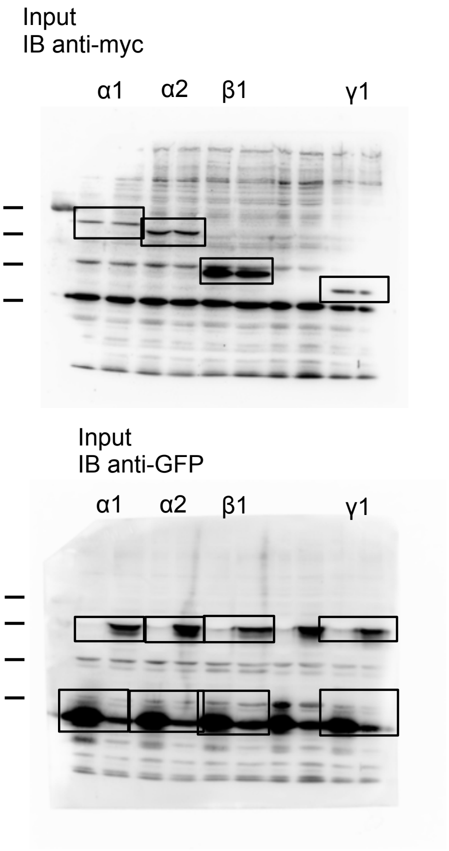

Supplementary Figure 8c

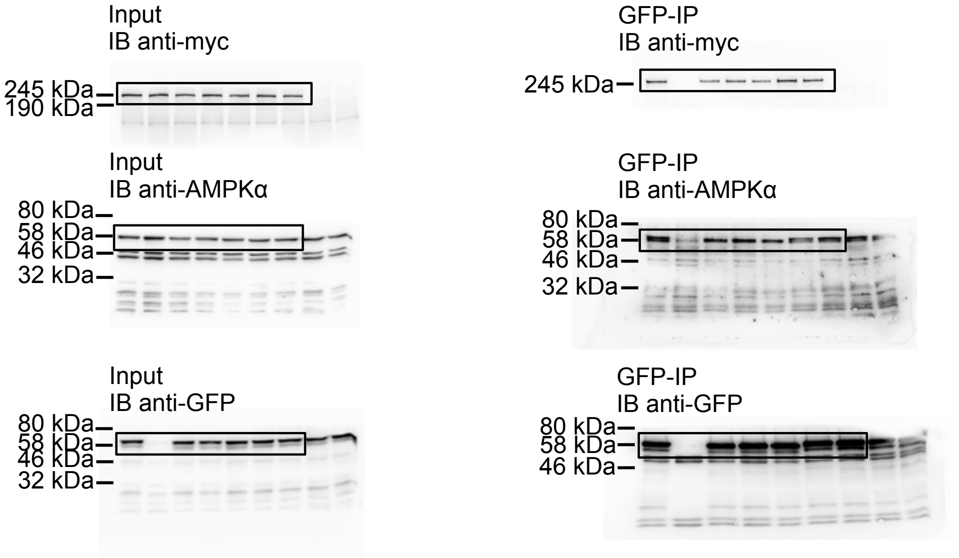

Supplementary Figure 9 Uncropped immunoblotting scans. For each immunoblot shown in Figures 1b, 2f, 4a, 5a, 5b, 5c, 5d, 5e, 5f, 5i, 5j, 6a, 6d, 7a, 7b, 7f, 7g, 7h, 7j, 7k, 8b, 8d and Supplementary Figures 1b, 1c, 1d, 3a, 3b, 4, 7a, 7b, 7c, 7d, 8c, uncropped scans are provided along with the location of molecular weight markers. Cropped sections presented in the corresponding Figures and Supplementary Figures are boxed. IB: immunoblot; IP: immunoprecipitation.

SUPPLEMENTARY NOTE 1

WIPI3 variants employed in this study

Initially, we employed our original WIPI3 cloning isolate[1] (referred to as WIPI3S in the Supplementary Figure 1), but structural homology modelling revealed that WIPI3S lacked the potential to fold into a 7-bladed β-propeller protein and was unable to bind to phosphoinositides (Supplementary Figure 1b). Based on these findings, we assessed an additional WIPI3 cloning isolate that encoded an N-terminal extended WIPI3 protein sequence (Supplementary Figure 1a). This N-terminal extended version of WIPI3 indeed folds into a 7-bladed β-propeller protein (Figure 1a) and specifically binds PtdIns3*P* and PtdIns(3,5)P_2 (Figure 1b, Supplementary Figure 1b, d), a hallmark feature of the PROPPIN family[2]. In support of this observation, site-directed mutagenesis of two conserved arginine residues critical for phosphoinositide binding in PROPPIN members[2] abolished the binding of the WIPI3-R230A/R231A mutant to PtdIns3*P* and PtdIns(3,5)P_2 (Supplementary Figure 1c). As expected, endogenous WIPI1, WIPI2 and WIPI4 (Figure 1b, left panels; Supplementary Figure 1d), along with their GFP-tagged variants (Supplementary Figure 1d), bound PtdIns3*P* and PtdIns(3,5)P_2. GFP-WIPI3 also bound PtdIns3*P* and PtdIns(3,5)P_2 (Figure 1b, left panel; Supplementary Figure 1b-d).

SUPPLEMENTARY REFERENCES

1. Proikas-Cezanne, T. *et al*. WIPI-1alpha (WIPI49), a member of the novel 7-bladed WIPI protein family, is aberrantly expressed in human cancer and is linked to starvation-induced autophagy. *Oncogene* **23**, 9314-9325 (2004).
2. Thumm, M. *et al*. It takes two to tango: PROPPINs use two phosphoinositide-binding sites. *Autophagy* **9**, 106-107 (2013).
